# Optimization of Cell Membrane Purification for the Preparation and Characterization of Cell Membrane Liposomes

**DOI:** 10.1002/smtd.202400498

**Published:** 2024-10-21

**Authors:** Sander de Weerd, Emma A. Ruiter, Eleonora Calicchia, Giuseppe Portale, Jan Jacob Schuringa, Wouter H. Roos, Anna Salvati

**Affiliations:** ^1^ Nanomedicine and Drug Targeting Groningen Research Institute of Pharmacy University of Groningen A. Deusinglaan 1 Groningen 9713 AV The Netherlands; ^2^ Molecular Biophysics Zernike Institute University of Groningen Nijenborgh 4 Groningen 9747 AG The Netherlands; ^3^ Department of Experimental Hematology, University Medical Center Groningen University of Groningen Groningen 9700 RB The Netherlands; ^4^ Macromolecular Chemistry & New Polymer Materials, Zernike Institute University of Groningen Nijenborgh 4 Groningen 9747 AG The Netherlands

**Keywords:** cell membrane purification, cell membrane liposomes, leukemia, targeted nanoparticle

## Abstract

Cell membrane nanoparticles have attracted increasing interest in nanomedicine because they allow to exploit the complexity of cell membrane interactions for drug delivery. Several methods are used to obtain plasma membrane to generate cell membrane nanoparticles. Here, an optimized method combining nitrogen cavitation in isotonic buffer and sucrose gradient fractionation is presented. The method allows to obtain cell membrane fractions of high purity from both suspension and adherent cells. Comparison with other common methods for membrane extraction, where mechanical lysis using cell homogenizers is performed in isotonic or hypotonic buffers, shows that the optimized procedure yields high purity membrane in a robust and reproducible way. Procedures to mix the purified membrane with synthetic lipids to obtain cell membrane liposomes (CMLs) are presented and indications on how to optimize these steps are provided. CMLs made using crude membrane isolates or the purified membrane fractions show different uptake by cells. The CMLs made with the optimized procedure and liposomes of the same composition but without cell membrane components are thoroughly characterized and compared for their size, zeta potential, bilayer and mechanical properties to confirm membrane protein inclusion in the CMLs. Cell uptake studies confirm that the inclusion of membrane components modifies liposome interactions with cells.

## Graphical abstract

This work presents a detergent‐free workflow based on nitrogen cavitation in isotonic buffer and sucrose gradient fractionation for the extraction and purification of cell membranes and preparation of cell membrane liposomes (CMLs). Multiple methods are combined to confirm the purity of the isolated membranes and to characterize the obtained CMLs.

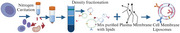

## 1 Introduction

Cells communicate with each other via complex interactions mediated at the cell membrane. Exploiting cell membrane interactions can enable novel ways to design nanomedicines for drug delivery. Synthetically replicating the complexity of cell membranes is exceptionally difficult. Because of this difficulty, biomimetic nanoparticles including cell membrane extracts and components have become an emerging tool in nanomedicine due to their unique ability to engraft cell‐like functionalities onto nanoparticles.^[^ ^1^, ^2^, ^3^, ^4^ ^]^ Contrary to synthetic bottom‐up approaches, which pose complex bio‐engineering challenges to conjugate specific biological functions onto nanoparticles, cell membrane nanoparticles use the full repertoire of glycogens, proteins, antigens, and lipids present in the plasma membrane to prepare novel nanomaterials, offering unique opportunities for tool‐development and nanomedicine‐targeting.

This top‐down approach has been successfully used to address some of the current long‐standing challenges in nanomedicine delivery, and in particular to camouflage nanoparticles as “self”, thus reducing their clearance by the immune system, or to promote targeting for drug delivery applications.^[^ ^5^, ^6^ ^]^ For instance, cell membrane nanoparticles were generated using red blood cell membranes to evade clearance by the immune system.^[^ ^7^ ^]^ In this case, the presence of the protein CD47 (cluster of differentiation 47) in the membrane of red blood cells prevented uptake by macrophages.^[^ ^3^ ^]^ Conversely, the predominant uptake of nanoparticles by immune cells was exploited with nanoparticles made from tumor cell‐membrane, in order to elicit an anti‐tumor response from the immune system.^[^ ^4^, ^8^ ^]^ Similarly, leukocyte membrane particles have been used to equip nanoparticles with the leukocytes’ ability to traverse the endothelium^[^ ^9^ ^]^ and to specifically target inflamed tissue.^[^ ^10^ ^]^ In other examples, stem cell membranes were used to make cell membrane liposomes (CMLs) and exploit their capacity to cross the blood–brain barrier, as well as to target cells for drug delivery.^[^ ^11^, ^12^ ^]^ Although cell membrane nanotechnology offers numerous possibilities, challenges in the generation and design of CMLs still persist.

For instance, there are huge differences between the reported methods to purify the biological membranes needed for the preparation of cell membrane nanoparticles.^[^ ^1^ ^]^ For a long time, extraction was the standard. Commercial kits to solubilize proteins from the membrane using detergents are available. However, these detergents can prove difficult to remove and can cause problems in the evaluation and stability of the cell membrane nanoparticles.^[^ ^10^ ^]^ Furthermore, weakly bound lipids, cofactors, and protein‐protein interactions are undoubtedly lost in the extraction procedure.^[^ ^13^ ^]^ On the other hand, there are methods that use detergent‐free buffers to isolate cell membranes, either in isolation or paired with a delicate physical disruption step.

In this work, we propose an adaptable framework that combines nitrogen cavitation and sucrose gradient fractionation for the isolation and purification of cell membranes. Nitrogen cavitation is used as a gentle lysis method in isotonic buffer to leave internal organelles intact and to separate them from the plasma membrane in order to obtain a crude membrane fraction. Thereafter, a sucrose density fractionation is used to further separate mitochondria and other internal membranes from the plasma membrane material and obtain a purified plasma membrane fraction. The results obtained with this method are compared to those obtained with standard mechanical lysis with a cell homogenizer and with hypotonic buffer. The composition and purity of the isolated cell membrane are characterized by western blotting and mass spectrometry. The results show that with the optimized method, the final product consists of ≈60% plasma membrane‐associated proteins by mass. The same workflow is applied to a different cell line to demonstrate that the method can be transferred to both cells that grow in suspension and in adhesion. We also show that CMLs made using a crude membrane fraction or the purified membrane show different behavior on cells, thus the level of purity of the extract affects the CML properties.

Thereafter, we discuss methods to mix the isolated cell membranes with synthetic lipids to obtain cell membrane liposomes and crucial aspects and steps in these procedures that require optimization, such as, for instance, the choice of the synthetic lipids used for inclusion of the purified membranes into CMLs.

We then use a combination of methods, including dynamic light scattering (DLS) and zeta potential measurements, small angle X‐ray scattering (SAXS), and atomic force microscopy (AFM) imaging to characterize the optimized CML physico‐chemical properties and compare them to those of liposomes of the same composition, but without cell membrane components. The detailed physico‐chemical characterization confirms that the inclusion of cell membrane components changes the bilayer properties substantially. Finally, to assess the biological functionality of the CMLs obtained with the optimized workflow, flow cytometry, and fluorescence microscopy are used to quantify and visualize their accumulation into different cell types. Results obtained for multiple extractions and multiple batches of CMLs are included to confirm the robustness of the procedure and reproducibility of the obtained CMLs. Overall, the presented methods and workflow offer a flexible and comprehensive approach for the extraction and purification of cell membranes, and for their inclusion into CMLs and their characterization.

## 2 Results and Discussion

### 2.1 Cell Lysis and Preparation of Plasma Membrane Vesicles

**Figure 1:**
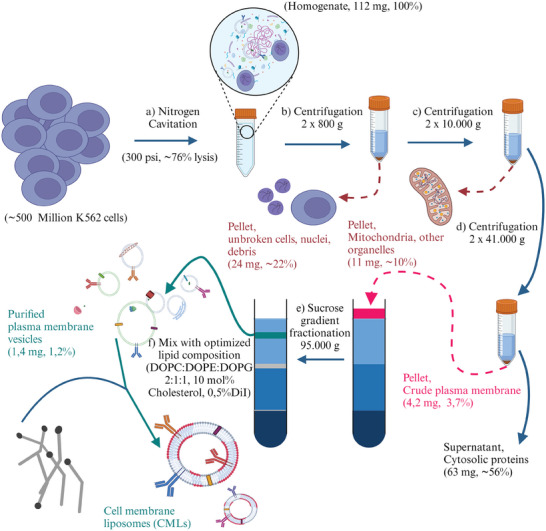
Schematic representation of the workflow for the generation of cell membrane liposomes (CMLs) after cell lysis by nitrogen cavitation. The optimized conditions and protein yields (mg and %) obtained for K562 cells are included. The average mass of proteins recovered is indicated. The percentages show the corresponding fraction for each pellet or supernatant, with respect to the total mass of proteins in the total homogenate. The mean and standard deviation of the results obtained in 5 independent extractions from around 500 million cells are shown (*n* = 5, see Table 1 for results of individual experiments). a) Gentle lysis of cells with nitrogen cavitation. b) Removal of nuclei and residual intact cells. c) Removal of mitochondria. d) Pelleting of crude membrane. e) Separation of internal membranes from plasma membrane based on density using sucrose gradient fractionation. f) Inclusion of synthetic lipids (for K562 cells: DOPC:DOPE:DOPG 2:1:1 with 10 mol% cholesterol and 0.5% DiI) to dilute cell membrane vesicles and generate monodisperse CMLs. Created with BioRender.com.

Human leukemia K562 cells were used to optimize the proposed methodology and workflow. Ideally, cell membranes should be isolated in a way that allows native biological molecules and cell membrane proteins to be maintained in their natural environment. This is because certain proteins rely on the presence of specific membrane lipids to function properly, and extraction procedures to purify solely membrane proteins may compromise protein structure and functionality.^[^ ^14^ ^]^ When cell membranes are isolated, the cell membrane fragments usually spontaneously re‐arrange into plasma membrane vesicles or stay as fragments, which then need to be purified from other potential intracellular membranes and components, prior to generating cell membrane nanoparticles. An overview of the involved steps is shown in **Figure** **1**, together with the yields obtained in the different steps in the case of K562 cells. To start with, a sufficient number of cells needs to be lysed. This is a well‐known bottleneck in cell membrane nanotechnology, as typically more than 200–300 million cells are required, which can limit application.^[^ ^1^ ^]^ Methods to reduce this number and increase yield are highly sought to push this technology further. Importantly, however, the level of purity of the isolated membranes strongly affects the biological properties of the resulting nanoparticles, as we explicitly test and show further in this work. Thus, procedures that may lead to a higher yield of a less pure isolate should be used with caution, and overall strict controls to test the purity of the isolate are an important step in any of these extraction procedures, as illustrated further.

The first step in the procedure is to break the plasma membrane to release the cells’ internal membranes and soluble proteins (Figure 1a). A crucial aspect in the extraction procedure is that internal membranes should remain intact to avoid contamination of other intracellular components, especially intracellular membranes. The disrupted cell membrane will stay fragmented or self‐assemble into cell membrane vesicles with their respective proteins.^[^ ^13^ ^]^ Residual intact cells and intracellular organelles, such as nuclei and mitochondria, are larger than cell membrane vesicles and can be separated using differential centrifugation (Figure 1b,c). Then, a crude membrane fraction is pelleted, leaving most cytosolic proteins and macromolecular complexes in the supernatant (Figure 1d). As a next step, cell membrane vesicles should be further separated from other intracellular membranes and smaller organelles. This can be difficult since the plasma membrane contributes to less than 10% of the total membranes present.^[^ ^15^ ^]^ Internal membranes have usually different densities than the cell membrane vesicles, allowing for separation using density fractionation (Figure 1e). As mentioned, procedures skipping this step may allow higher yield at the expense of purity of the isolated materials. Finally, cell membrane vesicles can be fused with synthetic lipids to generate CMLs (Figure 1f).

To break the cells, we evaluated and compared nitrogen cavitation with manual glass homogenization with a Potter‐Elvehjem homogenizer in isotonic buffer, as well as extraction in hypotonic buffer. All these methods allow for gentle lysis and introduce minimal mechanical force into the system.^[^ ^16^ ^]^ For purification purposes, we used a combination of differential centrifugation and sucrose density fractionation, based on a previously developed method by Suski and colleagues, that we modified specifically for gentle lysis using nitrogen cavitation.^[^ ^17^ ^]^

#### 2.1.1 Manual Homogenization

For manual homogenization, cells were resuspended at ≈10^7^ cells mL^−1^ in isolation buffer made of Tris, mannitol, sucrose, EGTA, MgCl_2_, and protease inhibitors, and lysed using a Potter–Elvehjem homogenizer on ice. Additionally, cells were also resuspended and lysed in hypotonic buffer, consisting of the same constituents but without mannitol and sucrose. Mechanical lysis with a homogenizer can be difficult to control and reproduce, because the efficacy of the method is heavily operator‐dependent, as we have also observed. Six cycles of 30 strokes of the homogenizer were needed to obtain a lysis percentage over 40% using isotonic buffer (**Table** **1**). This decreases the potential yield of plasma membrane considerably, especially when a very large number of cells are processed. The use of hypotonic conditions facilitates lysis, thus, in this case, 3 cycles of 30 strokes were sufficient to obtain over 80% lysis (Table 1). Another factor affecting methods based on manual homogenization is that the disruption process, including centrifugation, can take several hours. Over time, internal membranes such as those from mitochondria and lysosomes can start to leak. Eventual release of their contents can damage proteins or make it more difficult to separate them. Additionally, when hypotonic buffers are used, because of the membrane destabilization in these conditions, proteins can be lost from the extracted membranes and often sucrose needs to be added to limit this and improve membrane fragment stability.^[^ ^18^ ^]^ Therefore, to produce plasma membrane vesicles in a faster and more reproducible way, and to limit potential damage and similar loss of proteins in the procedure, nitrogen cavitation in isotonic buffer was explored as an alternative extraction procedure.

**Table 1:** Strokes, pressures, lysis percentage, and yield of plasma membrane extractions from K562 and MS5 cells performed using a Potter–Elvehjem homogenizer in isotonic and in hypotonic buffers and nitrogen cavitation in isotonic buffer, with and without magnesium chloride. The results obtained in different optimization experiments are shown. For the extractions by cavitation from K562 cells, the results obtained with the optimized settings in 5 independent extractions are shown, together with their average and standard deviation (mean ± SD, *n* = 5). The same average results for the optimized settings are indicated in the scheme of Figure 1.

Plasma membrane extraction by potter‐elvehjem homogenizer and nitrogen cavitation					
MgCl_2_	Strokes	10^6^ K562 cells	Lysis %	Crude membrane protein yield	Purified membrane protein yield
Potter‐Elvehjem homogenizer in hypotonic buffer – K562 suspension cells					
2.5 mm	3 × 30	624	85%	1.6 mg	0.7 mg
Potter‐Elvehjem homogenizer in isotonic buffer – K562 suspension cells					
2.5 mm	6 × 30	624	44%	1.1 mg	0.4 mg

Nitrogen cavitation – K562 suspension cells					
MgCl_2_	Pressure (psi)	10^6^ K562 cells	Lysis %	Crude membrane protein yield	Purified membrane protein yield
No	200	200	59%	1.8 mg^a)^	
No	300	500	92%	0.3 mg^b)^	
2.5 mm	250	76	72%	0.5 mg	
2.5 mm	300	84	79%	0.3 mg	
2.5 mm	350	97	86%	0.8 mg	
Final optimized settings for nitrogen cavitation – K562 suspension cells					
2.5 mm	300	470	76%	3.9 mg	0.9 mg
2.5 mm	300	400	80%	2.5 mg	0.5 mg
2.5 mm	300	470	74%	3.2 mg	1.0 mg
2.5 mm	300	660	79%	5.5 mg	1.9 mg
2.5 mm	300	580	71%	4.7 mg	1.5 mg
Average results (mean ± SD)		**510 ± 100**	**76% ± 4%**	**4.2 ± 1.6 mg**	** 1.4 ± 0.9 mg**

Nitrogen cavitation – MS5 adherent cells					
MgCl_2_	Pressure (psi)	10^6^ MS5 cells	Lysis %	Crude membrane protein yield	Purified membrane protein yield
2.5 mm	125	100	61%	1.5 mg	
2.5 mm	150	100	76%	2.0 mg	1.7 mg^c)^
2.5 mm	175	100	82%	2.0 mg	
2.5 mm	200	110	90%	2.0 mg	
2.5 mm	300	110	99%	1.4 mg	
2.5 mm	200	220	91%	3.4 mg	1.3 mg
2.5 mm	200	290	83%	3.5 mg	1.0 mg
2.5 mm	250^d)^	250	72%	3.8 mg	1.6 mg
2.5 mm	280^d)^	180	84%	2.1 mg	0.6 mg

^a)^ No 2 × 10000 g step before obtaining crude;

^b)^ No 2 × 10000 g step before obtaining crude and crude obtained by centrifugation at 27000 g instead of 41000g;

^c)^ Combined crude of 150, 175, and 200 psi for sucrose gradient purification;

^d)^ Cells collected via scraping in PBS instead of 2 mm EDTA in PBS and scraping to detach cells.

#### 2.1.2 Nitrogen Cavitation

Cavitation has previously been shown to be faster and more reproducible than manual homogenization.^[^ ^19^ ^]^ Briefly, cells in isolation buffer are placed in a metal vessel, and pressurized using N_2_ gas. When the pressure is released, N_2_ will bubble out of the solution sending pressure waves through the suspension that break the cells. An additional benefit of this method is that the expansion of gas is an endogenous process, taking up heat from the environment, contrary to most other methods of cell disruption that instead introduce heat into the system, such as with manual homogenization (as a consequence of force).^[^ ^20^ ^]^ A complete step‐by‐step protocol for membrane extraction via cavitation is given in Protocol S1 (Supporting Information). Previously, in our group, we found that using manual homogenization, a lysis percentage of 80% minimizes damage to internal organelles, which could contaminate the isolated fraction.^[^ ^21^ ^]^ The same was found to be true when disrupting mammalian cells using cavitation.^[^ ^22^ ^]^ Of note, for the purpose of cell membrane isolation, in the case of adherent cells that cannot be detached by scraping, EDTA and/or scraping should be preferred over trypsin for detaching cells, since trypsin can damage the membrane proteins. When scraping alone is not sufficient, EDTA can be used to detach cells adhering more strongly (Table 1). However, EDTA can promote the loss of membrane proteins anchored with divalent cations. A simple solution to avoid protein loss during extraction, which can benefit membrane extraction for both adhering and suspension cells, is to add MgCl_2_ in the isolation buffer to saturate EDTA and stabilize internal membranes.^[^ ^23^, ^24^ ^]^ The harvesting method can affect the pressure needed to disrupt the cells as well: when MgCl_2_ is added, a higher pressure is needed to lyse cells (Table 1). Therefore, several pressures were tested and MgCl_2_ was also evaluated as an additive to the isolation buffer to stabilize (internal) membranes (Table 1).^[^ ^22^ ^]^ A similar optimization of the pressure and use of MgCl_2_ was required for transferring the method to other cells, in this case, MS5 adherent stromal cells. Every cell type may require a different pressure depending on the method used for harvesting or due to different intrinsic membrane stability.

##### Magnesium Chloride for Nitrogen Cavitation

When an isotonic buffer with sugar and mannitol for protein and membrane stability was used at 300 psi, 92% lysis was observed and only 0.3 mg of protein was found in the **crude membrane fraction (**as shown in Table 1). A crude membrane fraction consists of a mixture of membranes including, but not limited to, lysosomes, endosomes, endoplasmic reticulum (ER), leftover mitochondria, nuclei, and plasma membrane (Figure 1). The high lysis percentage indicated that cavitation was, indeed, successful. However, centrifugation at 27000 g did not afford sufficient crude membrane (Table 1). Possibly, the cell membrane and internal compartments were so shattered when reaching such high lysis percentages, that the self‐assembled plasma membrane vesicles were too small to pellet.^[^ ^25^ ^]^ Another possibility to explain the low yield is that the now free‐in‐solution large sticky strands of negatively charged DNA functioned as a net to pellet other micro‐sized objects during centrifugation. DNAse, RNAse, and benzonase were used for MS5 extractions in order to counteract this by shearing the DNA and RNA, thus reducing the viscosity and potentially increasing protein yield.^[^ ^26^ ^]^ Small divalent cations, such as MgCl_2_ are commonly used to hinder protein loss during extraction by conferring stability to (nuclear) membranes, preventing breaking and mixing with other membranes.^[^ ^1^ ^]^ Consistent with this, by adding 2.5 mm MgCl_2_, as well as by increasing the crude fraction centrifugation speed to 41000 g, we were able to increase the yield considerably (Table 1).

#### 2.1.3 Finding the Optimal Lysis Pressure for Cells

**Figure 2:**
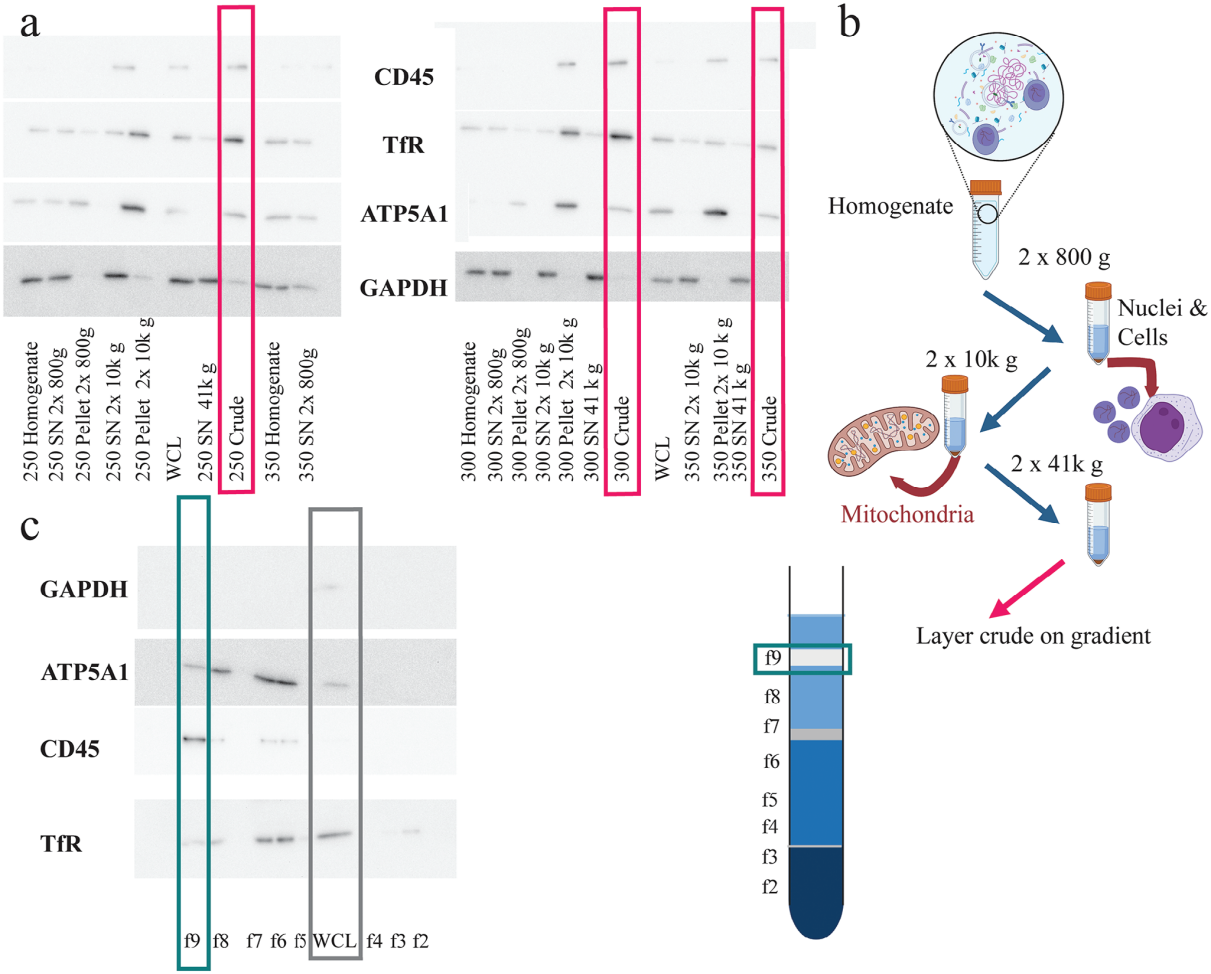
Western blots of the fractions obtained in the cell membrane extraction procedure for K562 cells. In Figure 2a,c the results for the crude fractions are shown in red boxes, and for the purified cell membrane fraction in green boxes. a) Western blots of fractions obtained after lysis using nitrogen cavitation at several pressures. Five micrograms of protein were loaded on each lane. Proteins were detected with antibodies against the cell membrane proteins cluster of differentiation 45 (CD45) and transferrin receptor (TfR), the mitochondrial membrane protein ATP synthase subunit a1 (ATP5A1), and the cytosolic marker glyceraldehyde‐3‐phosphate dehydrogenase (GAPDH). b) Schematic overview of the procedure and the supernatants and pellets evaluated by western blot. Created with BioRender.com. c) Western blot of the fractions obtained by sucrose gradient using the optimized protocol, stained with the same antibodies as in panel a. In all panels, the control used is a whole cell extract (WCL, grey box) obtained using a standard RIPA lysis protocol. The western blots obtained in one representative experiment are shown (comparable results were obtained in repeated extractions with the same procedure).

As mentioned earlier, the optimal pressure to lyse cells using cavitation differs between cells.^[^ ^20^ ^]^ Therefore, in order to optimize the protocol, ≈80 million K562 cells were lysed at several pressures, namely 250, 300, and 350 psi, in the presence of 2.5 mm magnesium chloride and purified, up till the crude membrane fraction was obtained (**Figure** **2**a,b). Several pressures were tested also for the lysis of MS5 cells (Figure S1, Supporting Information). As expected, for both cell types, the lysis efficacy increased when increasing the cavitation pressure (Table 1). Subsequently, the composition of pellets and supernatants (SN) was assessed by western blot for plasma membrane, cytosolic, and mitochondrial markers (Figure 2a; Figures S1 and S2, Supporting Information). To improve the purity of the crude product, centrifugation at 10000 g was shown to be very effective in removing the rather large (0.5–1 µm) mitochondria from the homogenate (Figure 2a). This indicated that mitochondria stay mostly intact during the cavitation procedure, resulting in their facile separation (Figure S2, Supporting Information). The crude fractions contained mostly membrane proteins and limited cytosolic proteins, as indicated by western blot analysis of relevant markers (Figure 2, red and green bars). Consistent with the observed lower protein yield (Table 1), at the highest lysis percentages fewer plasma membrane proteins were found in the crude fraction obtained from both MS5 and K562 cells (Figure 2a; Figure S1, Supporting Information). At 300 psi, the amount of plasma membrane markers was the highest. Therefore, for K562 cells, 300 psi was set as the optimal pressure for plasma membrane extraction. In the case of MS5 cells, the pressure used for the cavitation did not seem to influence the purity of the extract as much (Figure S1, Supporting Information), but influenced the yield (Table 1 – MS5). Hence, 200 psi was selected as the optimal pressure when EDTA was used for detachment. When recovering the cells by scraping instead, 280 psi was used (Table 1 – MS5).

In subsequent extractions, each time the procedure was repeated, ≈80% lysis was obtained with a yield ranging from 0.5 to 2 mg depending on the number and type of cells used, confirming that the method is robust and reproducible (Table 1).

Overall, the results show that with the inclusion of 2.5 mm MgCl_2_, nitrogen cavitation in isotonic buffer affords a fast and reproducible method for gentle cell lysis. The workflow presented can be easily transferred to other cells and can be used to optimize the conditions for the extraction of cell membranes from both adherent and suspension cells.

### 2.2 Purification of Crude Membrane Fractions by Sucrose Gradient

As mentioned earlier, many differences exist in the protocols used to isolate cell membranes, as well as the selection of the fractions used to generate cell membrane nanoparticles. In some cases, vesicles from a crude membrane fraction are directly used to generate functional particles.^[^ ^27^, ^28^ ^]^ In other cases additional purification steps are added. These further steps decrease the amount of material recovered, but allow obtaining a **purified cell membrane** fraction where other internal membranes are removed (Figure 2b,c).

In our case, we decided to include purification of the crude membrane fraction by centrifugation in sucrose gradient to then investigate the differences between crude and purified membrane fractions (Figure 2, crude fraction in red boxes, purified fraction in green) and test the effect of the further purification on the properties and interaction of the generated CMLs with cells.

#### 2.2.1 Crude Membrane Fraction Isolation and Characterization

**Figure 3:**
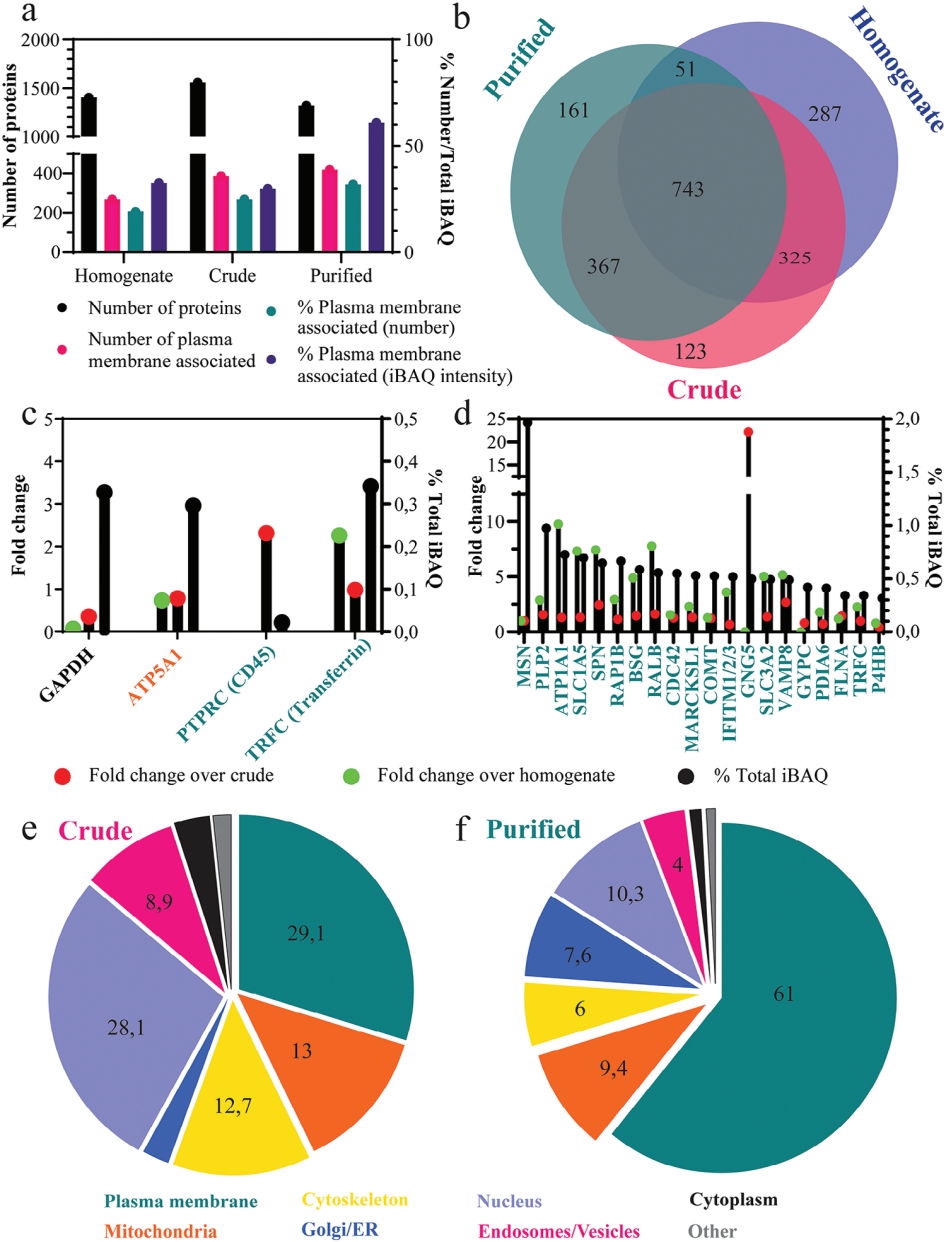
a) Proteomic analysis of the number and abundance (in respect to total protein abundance, as quantified by intensity‐based absolute quantification, iBAQ) of plasma membrane‐associated proteins in the homogenate, crude, and purified cell membrane fractions. b) Venn diagram of the number of proteins identified in the three samples. c) Fold changes and % of total iBAQ of proteins in the purified sample analyzed using proteomics, that were previously visualized with western blot for comparison. d) Fold change and relative abundance of most abundant plasma membrane proteins. e) Quantification of the plasma membrane protein percentages and proteins from other internal compartments of the crude and, f) purified products using Gene Ontology Cellular Component (GOCC) terms described in methods. The proteomic results of the fractions obtained in one representative extraction are shown (*n* = 1).

In order to isolate a crude membrane fraction from K562 extracts, nuclei can be removed by centrifugation at 3200 g, and by pelleting the crude membrane at 100000 g.^[^ ^8^, ^25^ ^]^ However, western blot analysis revealed that the crude membrane fraction obtained in such a way using cavitation included some cytosolic proteins (GAPDH), markers of plasma membrane (CD45, and the transferrin receptor TfR) and mitochondrial membrane components (ATP5A1) (Figure 2a,c red boxes). Additionally, the leftover supernatants at 41000 g appeared to be void of the integral cell membrane protein CD45, confirming that most of the cell membrane vesicles formed after cavitation were successfully pelleted into the crude membrane fraction (Figure 2). Not surprisingly, due to their similar sizes, others have found that the crude membrane fraction also contains other membranes including ER, Golgi, lysosomal, and mitochondrial membranes,^[^ ^17^, ^29^ ^]^ as we have also observed using proteomics (**Figure** **3**). Based on these findings, we set out to separate the internal membranes using density fractionation,^[^ ^17^ ^]^ which was previously shown to allow the purification of leukocyte plasma membrane for the generation of functional particles.^[^ ^9^ ^]^

#### 2.2.2 Purified Cell Membrane Fraction Using Sucrose Density Fractionation

To generate a purified membrane fraction, the crude membrane fraction was gently resuspended in a slightly acidic buffer (pH 6,0) including EDTA. Both are included to sever the intermembrane bonds that are being held together by protein‐protein interactions and divalent cations.^[^ ^24^ ^]^ Most MgCl_2_ needs to be removed by resuspension in MgCl_2_‐free buffer prior to this step, otherwise EDTA will be saturated. EGTA was used in the isolation buffer for its higher affinity to Ca^2+^ than Mg^2+^ for the purpose of inhibiting metalloproteases. Subsequently, the crude membrane was layered on top of a discontinuous sucrose gradient (52/43/38% w/w, leading to 9 fractions as depicted in Figure 2c), allowing for the separation of membranes based on density, instead of size. Western blot analysis of the obtained fractions showed that the cell membrane fragments (CD45, TfR) localized to the top of the 38% band (Figure 2c, green bar), while the denser mitochondrial membranes moved further down the gradient, toward the 43% and 38% interface (Figure 2c, f5 and f6). In accordance with previous work,^[^ ^9^ ^]^ the fraction with the highest amount of CD45 was found at the top of the gradient. This fraction was taken, diluted with buffer, and cell membrane vesicles were readily pelleted at 95000 g to remove the sucrose from the gradient. Cell membrane vesicles were then resuspended in a storage buffer with sucrose, mannitol, and protease inhibitors, aliquoted, and flash‐frozen in liquid nitrogen for long‐term storage at −80 °C.^[^ ^30^ ^]^ Western blot analysis confirmed that the purified membrane fraction contained a notable amount of membrane markers (TfR, CD45), minimal cytosolic proteins, and a lower mitochondrial contamination in comparison to the crude fraction (Figure 2c, green bar). Similar results were observed when following the same procedure for the purification of the MS5 cell crude fraction (Figures S1 and S2, Supporting Information).

Sucrose gradient purification and western blot analysis were performed also on the crude fractions obtained with the manual homogenization of K562 cells in isotonic and hypotonic buffers (Figure S3, Supporting Information). The results showed that these other methods also allow the purification of cell membranes, but in hypotonic buffer the protein yield is lower and a higher signal is observed for the mitochondrial marker in the final product in comparison to what was observed in isotonic buffer. More importantly, these methods require manual homogenization, which is time‐consuming and can be more difficult to reproduce among different operators. Instead, the presented workflow using nitrogen cavitation in isotonic buffer, followed by sucrose gradient fractionation allows the recovery of purified membranes with high yield and high purity in a fast, robust, and reproducible fashion.

#### 2.2.3 Proteomic Analysis of the Homogenate, Crude, and Purified Membrane

To further characterize the total homogenate, as well as the crude and purified fractions, mass spectrometry was performed using nanoLC‐MS/MS (liquid chromatography‐tandem mass spectrometry) on the crude and purified membranes obtained from K562 cells with the optimized method. Proteins were quantified using the label‐free quantification algorithm (iBAQ) incorporated in the MaxQuant software (Figure 3).^[^ ^31^ ^]^ The number of identified proteins was similar in the three samples (Figure 3a,b). When comparing the samples, the number of identified plasma membrane‐associated proteins (according to GOCC annotations plasma membrane and plasma membrane part) increased marginally in the crude and purified fractions in respect to the total homogenate, while the total quantity of plasma membrane‐associated proteins increased substantially (Figure 3a,e,f). This suggested the successful purification of cell membrane vesicles.^[^ ^32^ ^]^ Additionally, the proteomic data on CD45, TfR, ATP5A1, and GAPDH were consistent with the results obtained by western blot analysis for the same targets (Figure 3c) and showed a slight decrease in the mitochondrial and cytosolic content, while the plasma membrane share was increased as a result of the purification (Figure 2). Similarly, the most abundant plasma membrane‐associated proteins in the purified fraction were enriched in comparison to their abundance in the homogenate and crude fractions (Figure 3d). Out of the 21 proteins that showed the highest fold change in abundance in respect to their content in the homogenate, 13 were plasma membrane‐associated, 7 were proteins from other vesicles, 1 from mitochondria, and 1 from cytoplasm (Figure S4a, Supporting Information). Finally, 12 out of 20 most abundant unique proteins not found in the homogenate were plasma membrane proteins (Figure S4b, Supporting Information). Comparison of the protein content by cellular compartment using GOCC annotation and iBAQ quantification (see Experimental Section for details) confirmed a decrease in nuclei, endosomes/vesicles, and cytoskeleton in the purified sample compared to the crude fraction and a strong enrichment in plasma membrane proteins, consistent with the purification (Figure 3e,f).

This procedure can be translated by modifying the density gradient depending on the specific characteristics of the cells used. By using a similar combination of western blots of relevant markers and proteomics, the fraction containing cell membrane vesicles can then be identified and its purity verified. Similarly, the method can also be used to identify other fractions of interest (for instance other cellular organelles and membranes such as nuclei and mitochondria) and purify them for other applications.

### 2.3 Comparison of CMLs Obtained from Crude and Purified Membrane Fractions

#### 2.3.1 Effect of Membrane Purity on CML Properties and Cellular Interactions

**Figure 4:**
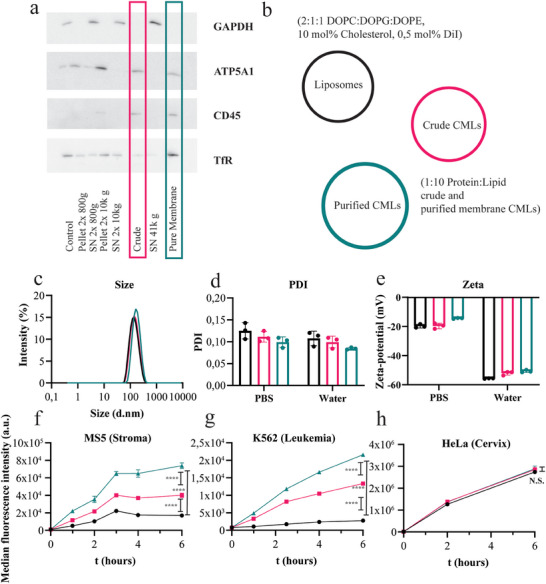
a) Western blot analysis of the different fractions obtained with the optimized protocol for membrane extraction and purification. In the boxes, the results for the crude (pink) and purified (green) fractions used for CML preparation are highlighted. 5 µg of protein was loaded on each lane, membranes were stained with GAPDH (cytosolic), ATP5A1 (mitochondrial), CD45 (plasma membrane), and TfR (plasma membrane). b) Schematic representation of the liposomes (black), crude (pink), and purified (green) CMLs evaluated in this figure. Their lipid composition and protein:lipid ratio are also indicated. c‐e) Size distribution (c), polydispersity index (PDI) (d) and zeta‐potential (e) in PBS of liposomes, crude CMLs, and purified CMLs (25 µg mL^−1^). In panels d and e, results in water are also included. f–h) Fluorescence intensity of MS5 cells (f) K562 cells (g) and HeLa cells (h) exposed for increasing time to 10 µg mL^−1^ liposomes and CMLs in 10% FBS. In panels (c–e), the results of 3 replicate measurements for a representative batch are shown, together with their average and standard deviation (mean ± SD, *n* = 3). The results in panels f‐h are the average and standard deviation over 3 replicate samples of the median cell fluorescence intensity measured by flow cytometry (mean ± SD, *n* = 3) in one representative experiment. An unmatched ordinary one‐way ANOVA with Tukey correction was performed to compare the cell fluorescence intensities at 6 h exposure. p < 0.05 was considered statistically significant. ****p ≤ 0.0001; N.S.: not significant In MS5 and K562 cells, CMLs show higher uptake in comparison to liposomes, whereas this is not the case for HeLa cells.

As a next step, the crude and purified cell membrane fractions were used to make CMLs and, in this way, explicitly test the effect of membrane purity on the properties and cell behavior of the resulting particles (**Figure** **4**). The results suggested that slightly larger nanoparticles were obtained when increasing the purity of the fraction used for particle preparation (131 nm for liposomes; 138 nm for CMLs obtained using the crude membrane faction and 160 nm for the purified fraction) (Figure 4c), with a slightly lower polydispersity index (PDI) (Figure 4d), and similar zeta potential, (−20 mV for liposomes and crude CMLs and −14 mV for purified CMLs (Figure 4e).

The uptake of liposomes, crude membrane CMLs, and purified membrane CMLs was evaluated in the presence of 10% FBS on the cells from which the particles were made of (human leukemia K562 cells), in murine bone marrow stromal MS5 cells known to interact with leukemia cancer cells, and in another cancer cell line for comparison (human breast cancer epithelial HeLa cells). When MS5 cells were exposed to K562 CMLs, an increase in nanoparticle uptake could be seen with respect to liposomes of the same composition but without membrane components. The increase was higher with increased membrane purity (Figure 4f). The same trend was observed when exposing K562 CMLs to K562 cells (Figure 4g). To verify whether the increase of uptake was cell‐type specific, and not due to other differences in particle properties, comparable uptake studies were also performed on HeLa cells (Figure 4h). Interestingly, HeLa cells showed no difference in uptake efficiency between the liposomes and the two different CMLs. These results suggested that the transplanted interactions originating from the K562 cells seemed to correlate with donor membrane purity and allowed to obtain higher uptake on cells known to interact with the parent cells, such as the stromal cells as well as the parent cells themselves (sometimes referred to as homotypic targeting).^[^ ^8^ ^]^

Given the observed effect of membrane purity on CML interaction with cells, only the **purified membrane** extracts were used for CML preparation for further experiments.

### 2.4 Additional Aspects and Steps in the Preparation of Cell Membrane Liposomes

#### 2.4.1 Selection of Synthetic Lipids to Add to Cell Membrane Vesicles for CML Preparation

**Figure 5:**
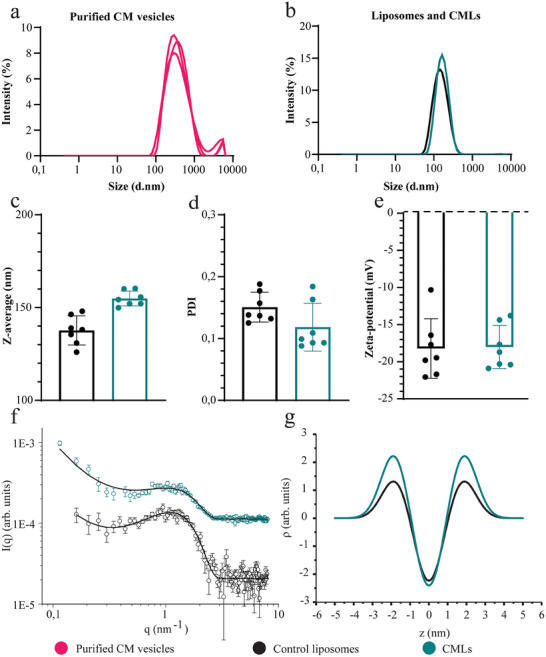
a) Size distribution of cell membrane vesicles (≈0.07 mg mL^−1^ protein in PBS) obtained after centrifugation at 95000 g and resuspension (corresponding to the purified fraction shown in the green box in Figure 2c) for three repeated measurements (*n* = 3). The results show minimal aggregation and a size distribution between 100 and 2000 nm. The vesicles have a zeta potential of ≈−9 mV. b) Size distribution of control liposomes and CMLs prepared by freeze‐thaw and extrusion using various membrane extracts (≈25 µg mL^−1^ in PBS). The average values of the distributions obtained for 7 independent batches generated with various membrane extract are shown (mean, *n* = 7). c) Z‐average (nm), d) PDI (a.u), and e) zeta potential (mV) of CMLs and control liposomes made with 10 mol% cholesterol and different plasma membrane extracts (≈25 µg mL^−1^ lipids in PBS). Each dot shows the average of 3 measurements performed in PBS for 7 independent batches, together with the average and standard deviation of the results obtained for all batches (mean ± SD, *n* = 7). The results confirm that the method allows to obtain CMLs and control liposomes with high reproducibility. f) Solution SAXS profiles of a representative batch of liposomes and CMLs at 2.5 mg mL^−1^ (*n* = 1, with the error bars corresponding to the experimental noise of the measured intensity, estimated after subtraction for the experimental blank background). Solid lines are the best fit obtained using the model for a single bilayer with the electron density profile described by a three Gaussian model.^[^ ^43^ ^]^ g) Derived electron density profiles for the bilayer across the membrane thickness for the control and CMLs.

The cell membrane vesicles obtained after sucrose gradient fractionation were characterized by DLS and zeta potential measurements after isolation. The results showed that they had a large size PDI (0.31), a Z‐average diameter of ≈320 nm, and zeta potential of −9 mV in PBS (**Figure** **5**a). These vesicles can, in principle, be used as they are or after an extrusion step or equivalent method to improve their size distribution. However, extruding purified cell membrane vesicles can be hard, because of the presence of high amounts of proteins and saturated lipids. For this reason, as well as to generate a larger amount of CMLs, dilution with synthetic lipids (including fluorescent lipids for labeling) is often used to allow further optimization of the final suspension and more control over particle size (Figure 5a). In this work, a 1:10 w/w protein to lipid ratio was used to ensure all vesicles included some membrane proteins,^[^ ^30^ ^]^ yet further dilutions have been used in the literature.^[^ ^10^ ^]^ However, for the purpose of transplanting specific interactions into a nanoparticle, diluting the membrane likely diminishes the chance to observe specific interactions, since some of these can be mediated by multiple proteins. Additionally, upon application in biological fluids such as in serum, the interactions with endogenous molecules may screen them. On the other hand, increasing the protein content to a 1:5 w/w protein‐to‐lipid ratio afforded particles with a much broader size distribution that were difficult to extrude (data not shown). Overall, depending on the cells used to extract cell membranes and the synthetic lipids added, the protein‐to‐lipid ratio can be optimized for specific applications.

#### 2.4.2 Choice of Synthetic Lipids

##### Phospholipids

The lipids used to prepare CMLs influence the size, zeta potential, and shape of the particles as well as the bilayer properties, thus they need to be carefully selected. It is known that some membrane proteins need specific lipids such as DOPE (dioleoylphosphatidylethanolamine) or cholesterol in order to function.^[^ ^30^, ^33^ ^]^ The plasma membrane of, for example, erythrocytes consists mostly of 1 part DOPC (dioleoylphosphatidylcholine, zwitterionic), 1 part sphingomyelin (zwitterionic), 1 part DOPE (zwitterionic), and 1 part anionic lipids such as DOPS (dioleoylphosphatidylserine) and between 20 and 50 mol% cholesterol.^[^ ^34^, ^35^, ^36^ ^]^ In this work, a comparable mix of synthetic lipids was chosen in order to mimic the natural environment of the proteins in mammalian cell membranes. Slightly different lipid mixtures were tested in order to define compositions that allowed to obtain stable CMLs of good phyisco‐chemical properties, as well as liposomes without membrane components but with comparable zeta potential. In this way, we could test the effect of the added membrane components on the obtained CMLs without confusing it with effects due to simple differences in charge. Table S1 (Supporting Information) summarizes all different compositions tested in this work. Additionally, a fluorescent lipid, DiI, was also included in order to obtain fluorescently labeled liposomes and CMLs and allow quantification of uptake by cells by flow cytometry and fluorescence microscopy.

As a first step, we evaluated different ratios of the zwitterionic lipids DOPC and DOPE to substitute for sphingomyelin (all zwitterionic) (Figure S5, Supporting Information). The mixture was 2:1:1 DOPC:DOPG:DOPE with 10 mol% cholesterol for stability and 0.5 mol% of the fluorescent lipid DiI for labeling. Other mixtures with more PE (PC:PG:PE 1:1:2) were also evaluated. The liposomes and CMLs obtained were tested in both MS5 stromal cells and the K562 used for cell membrane extraction. Cells exposed to control liposomes with higher PE content exhibited a broader uptake distribution by flow cytometry, suggesting heterogeneous behavior, which can complicate interpretation (Figure S5a,b, Supporting Information). In addition, uptake was higher for both control liposomes and CMLs when using higher PE content (Figure S5e,f, Supporting Information), possibly owing to the fusogenic nature of PE.^[^ ^37^ ^]^ Because of the differences observed, the mix with lower PE content was preferred.

##### Cholesterol and Stability

Another important component affecting the properties of the liposomes and whose amount needs to be optimized when preparing CMLs is cholesterol, which is usually used to stabilize liposomes. Compositions with 33 and 10 mol% cholesterol were investigated and, in this case, showed similar properties and stability over time (Figure S6, Supporting Information). The two lipid compositions exhibited similar uptake kinetics for up to 48 h in MS5 stromal cells (Figure S7a,b, Supporting Information). Similar results were found in K562 (Figure S5c, Supporting Information). Overall, both concentrations were suitable for the generation of liposomes and CMLs, and mixtures containing 10 mol% cholesterol were selected for further experiments. Of note, the particles were stored at 4 °C in filtered PBS with 0.05% sodium azide, and the stability of the different compositions was monitored over time (Figure S6, Supporting Information). The results showed that the particles were stable for up to 4 weeks after preparation.

#### 2.4.3 Mixing Membrane Extracts and Synthetic Lipids for CML Preparation

In order to allow the fusion of the cell membrane vesicles and synthetic lipids and generate liposomes of uniform properties and narrow size distribution, sonication, freeze‐thawing, and extrusion can be used. In these steps, care is key, as proteins can be compromised by repeated freeze‐thaw cycles and when using elevated temperatures. Sonication has the potential to disrupt the interactions between proteins, but ice baths and intermittent pulses can be used to mitigate the heat damage to proteins that may arise during sonication. Freeze‐thawing was shown to effectively fuse natural vesicles (exosomes) and liposomes.^[^ ^38^ ^]^ Furthermore, freeze‐thaw cycles are typically used to decrease the multi‐lamellarity of the final vesicles.^[^ ^39^ ^]^ Rapid cooling reduces the crystallization of water, which is the main reason for damage to proteins in repeated freeze‐thaw cycles. In our case, we used liquid nitrogen. Additionally, we chose to thaw the samples at room temperature (≈18–20°), rather than using a water bath at 37 °C, as is common for liposome preparation with this method, in order to keep samples above the phase transition temperature of the lipids, but not higher, and, in this way, limit the risk of protein damage. Hence, the optimized mix of unsaturated lipids, cholesterol, and fluorescent lipids for tracing was mixed in organic solvents and after evaporation of the solvent and thin layer formation, it was hydrated with a suspension of cell membrane vesicles to a final 1:10 w/w protein:lipid ratio. Afterward, samples were frozen in liquid nitrogen, thawed at room temperature 8 times, and finally extruded.^[^ ^38^, ^40^, ^41^ ^]^

#### 2.4.4 Extrusion

Extrusion is the preferred method for low‐scale CML preparation to control size.^[^ ^1^ ^]^ The mechanical force disrupts the membrane of the empty lipid vesicles and plasma membrane vesicles after which the fragments recombine. Given the presence of membrane proteins and the size of the starting cell membrane vesicles obtained after cell extraction (Figure 5a), first, a 400 nm polycarbonate (PC) membrane was used, followed by extrusion through a 200 nm PC membrane.

Silver staining of the proteins recovered in the particles after extrusion suggested a small reduction of protein intensity, proportional to the loss of fluorescence, but most bands were still present (Figure S8, Supporting Information). The lipid concentration of the CMLs after extrusion was estimated based on the concentration of the fluorescent lipid DiI which was included in the formulation. Even though this method does not allow for quantification of the real lipid concentration after inclusion of the cell membrane vesicles, the results suggested that almost all (≈80% over multiple batches) of the fluorescent lipid that was added to the cell membrane vesicles was recovered after extrusion (data not shown).

Characterization by DLS showed that the obtained CMLs were slightly larger than the control liposomes of the same composition without cell membrane components (Figure 5b,c) and because of the freeze and thaw cycles and the extrusion procedures, had low polydispersity. It is important to mention that given the small surface area of one liposome with respect to that of one cell, CMLs are expected to have heterogeneous properties in relation to the identity and amounts of the membrane components included in individual particles within a sample. High‐resolution single‐particle methods are required to characterize such heterogeneity among individual particles.

Multiple membrane extracts were prepared and used to prepare multiple batches of CMLs in order to test the reproducibility of both the extraction procedure and inclusion in CMLs. The results obtained confirmed that CMLs with comparable sizes and lower PDI could be obtained when using different membrane extracts, as well as when the same extract was used to prepare multiple CML batches (Figure 5b–d). This suggested that the optimized procedure was reproducible.^[^ ^10^ ^]^ Furthermore, the zeta potential of the liposomes and CMLs were almost identical (−18 mV) (Figure 5e). This is another advantage when using a detergent‐free lysis method to extract cell membrane, since detergents can interfere with the zeta potential of the final CMLs.^[^ ^10^ ^]^ As mentioned, the zeta potential has a strong effect on nanoparticle uptake. Because of this, having proper control liposomes with similar zeta potential, size, and composition of the CMLs, but without cell membrane components is important in order to be able to evaluate the differences in cell behavior due to the presence of membrane proteins, and not to confuse them with effects due to other physico‐chemical properties which may differ (such as zeta potential). The CMLs were stable in medium supplemented with 10% FBS as used for cellular studies (Figure S9, Supporting Information).

In conclusion, the optimized lipid composition to prepare CMLs with cell membrane extracted from K562 cells was set as **2:1:1 PC:PE:PG with 10 mol% cholesterol and 0.5 mol% DiI** (33 mol% cholesterol was also an option), and this composition allowed to obtain stable CMLs and liposomes of comparable properties.

### 2.5 Comparison of the Physico‐Chemical Properties of Optimized CMLs in Respect to Liposomes

#### 2.5.1 Effect of Membrane Inclusion on the Bilayer Structure Studied by Small Angle X‐Ray Scattering

After optimizing the inclusion of the purified membrane extract into CMLs, different methods were applied to characterize their physico‐chemical properties in order to determine how the inclusion of cell membrane components affects the bilayer and liposome properties.

First, the bilayer structure of liposomes and CMLs was investigated by solution small angle X‐ray scattering, SAXS (Figure 5f,g). This technique can provide information on the size and shape of the liposomes as well as on the arrangement of the lipid molecules in the membrane.^[^ ^42^ ^]^ Since the size of the liposomes is larger than the SAXS resolution, the SAXS profiles mostly provide information about the membrane structure. The absence of discrete peaks indicated that the liposomes were mostly unilamellar just after preparation, and we could exclude the presence of multilayered structures. The acquired SAXS curves could be well described using a widely accepted and simple model for a bilayered structure with Gaussian electron density profiles (EDP).^[^ ^43^ ^]^

The fitting provides the average bilayer thickness t and the distribution of the electron density across the membrane thickness, namely the electron density on the polar head side and in the inner part of the bilayer. The bilayer structural parameters obtained from the fit are summarized in Table S2 (Supporting Information). For the liposomes, we found a value of t = 3.6 nm, in agreement with previous reports on similar systems.^[^ ^44^ ^]^ For the CMLs, the recorded SAXS intensity was significantly altered. While the main broad intensity oscillation did not change in position dramatically (indicating that the bilayer thickness did not change significantly), the overall intensity increased significantly and the shape of the curve was altered. The overall increase in the scattered intensity is consistent with the incorporation of large integral membrane proteins inside the bilayer, while the change in the curve shape indicated changes in the electron density contrast of the bilayer. A comparison of the extracted EDPs showed that the composition of the liposome bilayer was mostly altered at the polar head side, while only a minor change was recorded for the inner core. This is consistent with the inclusion of peripheral membrane components. Furthermore, we also recorded an increased intensity at the highest angles (q > 3 nm^−1^) that can be explained by the presence of additional components such as proteoglycans, cytoskeletal components, and glycolipids transplanted from the cell membrane and not incorporated in the bilayer.

#### 2.5.2 Effect of Membrane Inclusion into the Bilayer Mechanical Properties Studied by Atomic Force Microscopy

**Figure 6:**
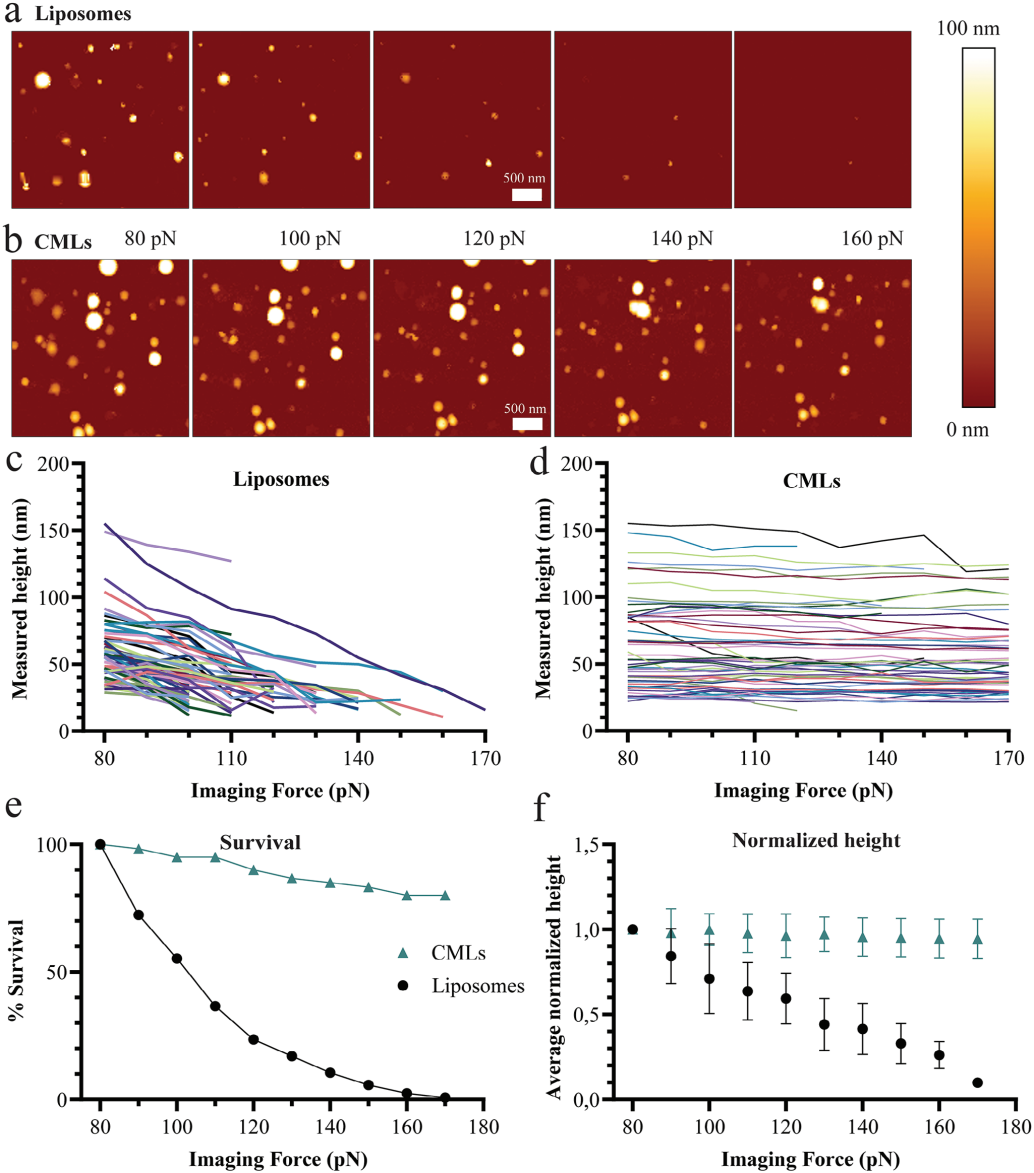
a,b) Examples of images obtained at different imaging forces for (a) liposomes and (b) CMLs. c,d) Heights of individual liposomes (c) or CMLs (d) in PBS immobilized on poly‐L‐lysine coated glass that sustained at least 3 consecutive imaging forces and were not multilamellar. e) Survival plot obtained from the data of panels c and d, after measuring 114 liposomes and 57 CMLs under the influence of increasing imaging forces (*n* = 114 and *n* = 57, respectively) in one representative experiment. The results show that respectively 1% of the liposomes and 81% CMLs survived. f) Normalized height data for the two nanoparticles under the influence of increasing imaging forces. The average of the individual results of panels c and d are shown after normalization, together with their standard deviation (mean ± SD, *n* = 114 and *n* = 57, respectively for liposomes and CMLs).

To further characterize the mechanical properties of the particles and confirm the insertion of membrane components in the bilayer, atomic force microscopy (AFM) was used. AFM is a powerful high‐resolution technique with the unique advantage of being able to probe the mechanical properties at single particle resolution. In order to achieve this, liposomes and CMLs were subjected to increasing imaging forces.^[^ ^45^, ^46^ ^]^ This was done in order to determine the relative deformability of the particles. Both liposomes and CMLs were negatively charged (Figure 5e) and could therefore be immobilized on poly‐L‐lysine coated glass and imaged under aqueous conditions according to previously published protocols.^[^ ^47^ ^]^ The softest available tip was used, and the starting pressure (80 pN) was chosen because it allowed to image the particles without excessive loss‐of‐contact events (more rigid tips might be used for coated spheres, or solid lipid nanoparticles). The Z height was picked to allow the tip to move freely, without dragging particles over the substrate. The 3 × 3 µm overview images show that the CMLs are more resistant to the applied pressure than the liposomes (**Figure** **6**a,b).

The imaging forces that were used for liposome and CML imaging ranged from 80 to 170 pN, with increments of 10 pN. Other imaging parameters were kept the same and the height is reported as a function of the imaging force (Figure S10, Supporting Information). The particles that sustained at least 3 consecutive pressures without breaking are shown in Figure 6c,d. For the liposomes, 58 out of 114 (51%) sustained these three consecutive pressures, whereas for the CMLs 54 out of 57 (95%) survived (Figure 6c–e). In a few cases, particles showed a decrease in height larger than 30 nm when imaged at the subsequent imaging force (10 liposomes and 1 CML, corresponding to <10% of the total 114 particles). Such a large drop in height can be observed for multilamellar liposomes, when the outer bilayer breaks, exposing an inner bilayer. These particles were excluded from the analysis because of their different mechanical properties (Figure S11a, Supporting Information).^[^ ^48^ ^]^ Overall, the fact that only a minor fraction of the imaged particles showed this behavior is consistent with the SAXS results which confirmed that the liposomes and CMLs were unilamellar.

When increasing the imaging force, all liposomes, except one, collapsed before being imaged at 170 pN, whereas 81% of the CML survived up until this force (Figure 6e). When plotting the normalized particle height as a function of the imaging force, a general decrease in height could be observed for the liposomes, which instead was much smaller for the CMLs (Figure 6f). The observation that the decrease in height is much smaller for the CMLs suggests that the CMLs are more rigid, because they are less susceptible to deformation upon increasing imaging force. The increased rigidity can be explained by the intercalation of membrane proteins and lipids into the bilayer. The single‐particle resolution of AFM also allowed us to appreciate the homogeneity of the particles: 80% of the CMLs behaved similarly until 170 pN, suggesting that, at least in this case, the imaged particles were all fusion products of cell membranes and synthetic lipids. In conclusion, the AFM data showed diverging rigidity between liposomes and CMLs, and these results, together with the increased size and decreased polydispersity observed by DLS, and differences in the SAXS profiles obtained for the two samples (Figure 5), suggested the successful incorporation of plasma membrane into the CMLs.

### 2.6 Interaction of CMLs and Liposomes with Cells

#### 2.6.1 Intracellular Distribution of Liposomes and CMLs

**Figure 7:**
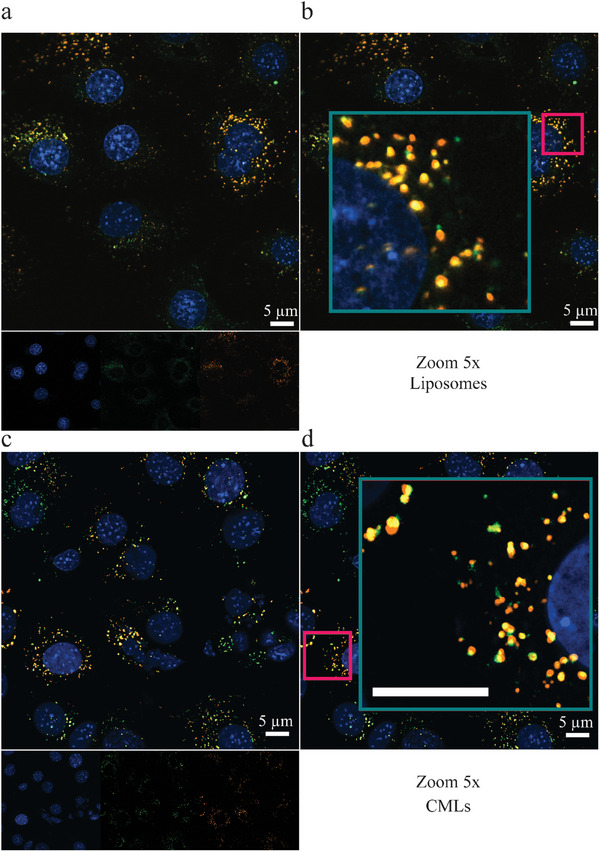
a) Live cell confocal image of MS5 cells exposed to 10 µg mL^−1^ a,b) liposomes or c, d) CMLs for 3 h in 10% FBS supplemented medium. Blue = Hoechst stained nuclei, green = LysoTracker green stained acidic compartments, and red = DiI (fluorescent lipid) labelled liposomes and CMLs. In b) and d) enlarged details of the images shown in a and c, respectively (5x zoom) (green), are shown. Scale bar: 5 µm. Brightness and contrast were optimized in ImageJ (Fiji). The results show that both CMLs and liposomes end up in the lysosomes.

As a following step, live cell confocal imaging was performed to confirm uptake and qualitatively analyze the intracellular distribution of liposomes and CMLs. Imaging was performed on stromal MS5 cells that were exposed for 3 (**Figure** **7**) and 24 h (Figure S12, Supporting Information) to both liposomes and K562 CMLs (Figure 7b,d). Generally, nanoparticles are internalized by endocytosis and end up in the lysosomes.^[^ ^49^, ^50^, ^51^ ^]^ Consistent with this, confocal microscopy confirmed the accumulation of both nanoparticles into the lysosomes, which was more evident at the longer exposure time, due to the higher amount of internalized nanoparticles.

#### 2.6.2 Uptake Studies in Bone Marrow Stromal Cells (MS5)

**Figure 8:**
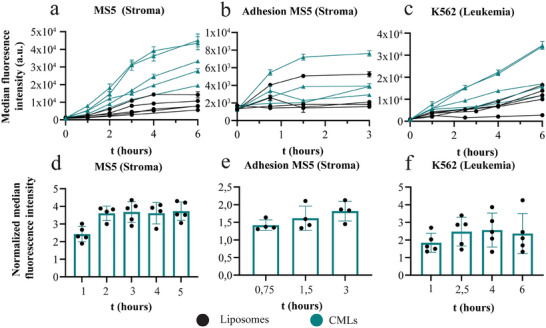
Uptake and adhesion of liposomes and K562 CMLs (10 µg mL^−1^ in medium supplemented with 10% FBS) in MS5 and K562cells. The particles used in these experiments were generated using at least 3 different membrane extracts and from various batches, on cells of varying passages. The reproducibility of the trends observed highlights the robustness of the presented method for membrane extraction and preparation of CMLs. The results are the average and standard deviation over 3 replicate samples of the median fluorescence intensity obtained by flow cytometry (mean ± SD, *n* = 3) in 5 (a,c) or 4 (b) independent experiments. From the data of panels (a–c), normalized data was obtained by dividing for each experiment the results obtained for CMLs by that obtained for control liposomes and are shown in panels (d–f) (mean ± SD; *n* = 5 for panels (d) and (f); *n* = 4 for panel (e).

As a final step, further uptake kinetic studies were performed in both stromal MS5 cells and the leukemia K562 cells from which the CMLs were made (**Figure** **8**). As shown in Table 1 and Figure 5, several extracts were generated and were used to fabricate a multitude of CML batches throughout the work. Figure 8 shows the cell uptake results obtained with these different batches, tested on cells of varying ages (passage numbers). Despite the many variables, such as cell age, particle aging, plasma membrane extract, and its quality (over time), the multiple particle batches and different operators, when MS5 cells were exposed to K562 CMLs and liposomes, nanoparticle uptake increased over time and was overall higher for CMLs. On average, uptake of the CMLs was 3.5‐fold higher than for liposomes after 2 h (Figure 8b). Depleting cell energy with NaN_3_ confirmed that the uptake of both liposomes and CMLs was active (Figure S13, Supporting Information). Uptake was higher for the CMLs also at longer exposure times (Figure S14, Supporting Information). To quantify the adhesion of CMLs and control liposomes to MS5 cells, cells were placed at 4 °C to prevent active internalization. After exposure, the particles in solution were washed away and the particles adhering to the cells were allowed to be internalized (by incubation at 37 °C for 3 more hours). The results showed that adhesion was roughly 1.5 times higher for CMLs (Figure 8b,e). Altogether, this data showed that bone marrow stroma cells have a higher uptake efficiency for K562 CMLs than for liposomes of similar physiochemical properties. The higher accumulation suggests that some of the interactions between the K562 cells and MS5 stromal cells are successfully transplanted onto the K562 CMLs and can be used to achieve higher uptake in these cells.

#### 2.6.3 Uptake Studies in K562 Cells

When K562 cells were exposed to K562 CMLs we observed similar results. K562 CMLs were taken up more by the cells they were derived from,^[^ ^8^ ^]^ whereas the control liposomes showed minimal uptake (Figure 8d). The difference increased gradually over time and after 2.5 h was ≈2.5 fold (Figure 8e). Higher uptake for CMLs with respect to the liposomes was observed also at longer exposure times of up to 30 h (Figure S15, Supporting Information). The higher uptake of K562 CMLs on the originating K562 cells may be explained by so‐called homotypic targeting behavior, where the cells preferably take up nanoparticles made from their own membrane when compared with control liposomes, likely due to self‐recognition proteins and adhesion proteins present in the CMLs.^[^ ^4^, ^8^ ^]^

## 3 Conclusion

Engineering CMLs allows to engraft cell‐like characteristics onto particles, which can be used to solve some of the long‐standing problems in nanoparticle development. The capacity to reconstitute pieces of cell membranes onto a particle can lead to the development of novel materials for drug delivery. There is a plethora of methods available for the extraction and purification of cell membranes, some of which use detergents that are difficult to remove and can cause downstream problems.

In this work, we present a fast, scalable, reproducible, and detergent‐free method for the extraction and purification of cell membrane and generation of high purity cell membrane liposomes from both suspension and adherent cells. The method is based on nitrogen cavitation in isotonic buffer, followed by purification in a sucrose gradient, and can be optimized and modified for other cells using the presented workflow. We also show that the purity of the cell membrane extract used for CML preparation affects the physiochemical properties and downstream biological interactions of the generated CMLs. We further discuss different parameters that need to be optimized for the preparation of CMLs, such as, for instance, the mix of synthetic lipids used for the incorporation of the membrane extract into liposomes. Having optimized the different steps in the procedure, we show that the obtained CMLs have the same zeta potential but are slightly bigger and have slightly lower PDI than liposomes of the same composition without membrane components. Furthermore, CMLs of comparable size, zeta potential, and cell uptake behavior are obtained when using different membrane extracts and for multiple batches, confirming that the procedure is robust and reproducible. SAXS results corroborate the inclusion of membrane proteins in the bilayer and AFM data indicate that the inclusion of membrane proteins and lipids conferred physical stability to the bilayer and that CMLs are more rigid than liposomes without cell membrane components. The optimized K562 CMLs show increased association and uptake in MS5 (bone marrow stromal cells), known to interact with leukemia cells, as well as the K562 cells from which they originated, but not in HeLa cells. Further studies are required to determine potential effects on the uptake of factors such as the nanoparticle mechanical properties or differences in the proteins they adsorb once in serum.^[^ ^46^ ^]^ Nevertheless, the different uptake behavior observed in the different cell types suggests that the transplanted interactions are cell‐type specific and that some of the specific niche interactions existing in vivo between leukemia cells with each other and with stromal cells can be recreated using cell membrane nanoparticles.

## 4 Experimental Section

### Cell Culture

K562 cells were purchased from DSMZ (Braunschweig, Germany) and cultured in RPMI 1640 with 2 mm GlutaMAX (Gibco, ThermoFisher Scientific, UK) supplemented with 10% fetal bovine serum (FBS, Gibco, ThermoFisher, Brazil), 2 mm L‐glutamine, 20 mm HEPES, in 37 C° with 5% CO_2_. The murine stomal (MS5) cells were purchased from DSMZ (Braunschweig, Germany) and were cultured in alpha mem medium (Gibco, ThermoFisher Scientific, UK) with 10% FBS in 37 C° with 5% CO_2_. HeLa cells were cultured in MEM (Gibco, ThermoFisher Scientific, UK) supplemented with 10% FBS. All cell lines were kept in culture for less than 20 passages and not used before 4 passages. Mycoplasma testing was performed regularly to exclude contamination.

### Lysis Percentage

The lysis percentage was determined by counting intact cells with a hemocytometer using reverse‐phase microscopy before and after cavitation.

### Manual Homogenization Using Potter–Elvehjem Homogenizer in Isotonic and Hypotonic Buffer

Isotonic starting buffer was prepared according to Protocol S1 (Supporting Information) with 225 mm mannitol, 75 mm sucrose, 30 mm Tris, pH 7.4. For the hypotonic starting buffer, sucrose and mannitol were omitted resulting in 30 mm Tris, pH 7.4. For both methods, 0.5 mm EGTA and 2.5 mm MgCl_2_ were freshly added according to Protocol S1 (Supporting Information) to obtain the isolation buffers. K562 cells were washed and collected in 1 falcon tube following steps 1–3. Then, cells were resuspended in 6–10 mL isotonic or hypotonic isolation buffer and placed in a 15 mL Potter–Elvehjem homogenizer with a tight‐fitting pestle. Thirty slow strokes on ice were performed after which the cells were centrifuged at 300 x g to collect the unbroken cells. This was repeated 3 times for the hypotonic buffer condition, and 6 times for the isotonic buffer. The supernatants were collected and pooled for plasma membrane isolation continuing the optimized method described in Protocol S1 (Supporting Information) from step 10.

### DC‐Bio Rad Protein Assay

Due to the high amount of lipids present in the samples, which may interfere with protein detection, a modified version of the Bio‐Rad DC protein assay was used, where SDS was added in order to improve the solubilization of the membrane proteins. Standards were prepared in the same buffer as used for the samples, with bovine serum albumin (BSA, 98% ThermoFisher Scientific, UK) at concentrations ranging from 0.0625 to 2 mg mL^−1^. The assay was performed according to manufacturers’ description but with the addition of 2% SDS to reagent B. Reagent S was also included because detergent was added to the assay. After 15 min absorbance was measured at λ = 650 nm using a ThermoMAX microplate reader. Afterward, protein concentrations in unknown samples were determined using the respective calibration curves.

### SDS‐Fel, Western Blot, and Silver Staining

SDS‐PAGE gels (10%) were prepared and loaded with 5 µg protein. When the concentration in the original sample was not high enough to reach 5 µg protein per lane, the undiluted sample was loaded. Then, proteins were denatured with 4x diluted loading buffer and heated to 95 °C for 10 min. Thirty‐five microliters of the diluted sample was loaded into the gel and run at 120 V. In the case of silver staining, the same volume of particles was loaded in the gels and after running, the gels were fixed with glacial acetic acid and stained. Gels were imaged in a ChemiDoc XRS (Biorad, USA).

For antibody detection, proteins were transferred to a CH_3_OH‐activated polyvinylidene fluoride (PVDF) membrane according to the manufacturers’ instructions for 1.5 h at 120 V in a cooled transfer tank. As a next step, the membrane was incubated in a blocking buffer (20 mm Tris‐base, pH 7.6, 1.5 M NaCl, 0.1% Tween‐20, and 5% non‐fat dry milk). Primary antibodies were added to the blocking buffer as follows: rabbit monoclonal anti‐GAPDH (1:2000, Cell signaling Technology, Catalog number: 51 745, Leiden, The Netherlands), mouse monoclonal anti‐CD45 (1:2000, Proteintech, Catalog number: 60287‐1‐Ig, USA), mouse monoclonal anti‐transferrin receptor (1:2000, Thermofisher Scientific, Catalog number: 13–6800), rabbit polyclonal anti‐ATP1A1 (1:2000, Proteintech, Catalog number: 55187‐a‐AP, USA), and rabbit polyclonal anti‐ATP5a1 (1:2000, Novus Biologicals, Catalog number: NBP2‐92928‐0.1 mL, USA). Secondary antibodies used for detection were: goat‐anti‐rabbit HRP conjugated secondary antibody (1:2000, Southern Biotech, Catalog number 4049‐05, USA) and rabbit‐anti‐mouse‐HRP conjugated (1:2000, Southern Biotech, Catalog number: 6160‐05, USA). The signal was detected using an ECL detection kit (GE Lifesciences) according to manufacturer instructions and imaged with a ChemiDoc XRS (Biorad, USA) after which the images were inverted with ImageJ, converted to 8‐bit with linear scaling, and placed in the panels with Adobe Illustrator 2022.

### Mass Spectrometry Analysis

Homogenate, crude, and purified protein samples (of similar mass) were loaded on an 8% precast RunBlue gel (Expedeon) and run at 100 V for 5 min. Gel staining was performed using InstantBlue (Expedeon) followed by a wash with ultrapure water. Comassie‐stained bands were excised in one gel slice and these were further cut into small pieces and destained using 70% 50 mm NH_4_HCO_4_ and 30% acetonitrile. The reduction was performed using 10 mm DTT dissolved in 50 mm NH_4_HCO_4_ for 30 min at 55 °C. Next, the samples were alkylated using 55 mm chloroacetamide in 50 mm NH_4_HCO_4_ for 30 min at room temperature and protected from light. Subsequently, samples were washed for 10 min with 50 mm NH_4_HCO_4_ and for 15 min with 100% acetonitrile. The remaining fluid was removed, and gel pieces were dried for 15 min at 55 °C. Tryptic digestion was performed by the addition of sequencing‐grade modified trypsin (Promega; 25 µL of 10 ng mL^−1^ in 50 mm NH_4_HCO_4_) and overnight incubation at 37 °C. Peptides were extracted using 5% formic acid in water followed by a second elution with 5% formic acid in 75% acetonitrile. The samples were dried in a SpeedVac centrifuge and dissolved in 20 µL 5% formic acid in water for analysis with LC‐MS/MS.

Samples were analyzed on a nanoLC‐MS/MS consisting of an Ultimate 3000 3000 RSLCnano system (Thermo Fisher Scientific, USA) interfaced with a Q Exactive plus mass spectrometer (Thermo Fisher Scientific). Peptide mixtures were loaded onto a 5 mm × 300 µm i.d. C18 PepMAP100 trapping column with 2% acetonitrile in 0.1% formic acid at 20 µL min^−1^. After loading and washing for 3 min, peptides were eluted onto a 15 cm × 75 µm i.d. C18 PepMAP100 nanocolumn (Thermo Fisher Scientific) held at 40 °C. A mobile phase gradient at a flow rate of 300 nL min^−1^ and with a total run time of 120 min was used: 2−30% of solvent B in 90 min; 30−80% B in 5 min, followed by a return to 2% B. Solvent A was water with 0.1% formic acid, and solvent B was acetonitrile with 0.1% formic acid. In the nanospray source, a stainless‐steel emitter (Thermo Fisher Scientific) was used at a spray voltage of 2 kV with no sheath or auxiliary gas flow. The ion transfer tube temperature was 250 °C. Spectra were acquired in data‐dependent mode with a survey scan at m/z 300−1650 at a resolution of 70000 followed by MS/MS fragmentation of the top 10 precursor ions at a resolution of 17.500. Singly charged ions were excluded from MS/MS experiments and fragmented precursor ions were dynamically excluded for 20 s.

### Database Annotation and Relative Quantification of MS/MS Data

MS/MS RAW files were processed with the MaxQuant software (version 2.1.0.0) using the Andromeda search engine for false discovery rate (FDR), controlled peptide and protein identification, and label‐free quantification. Label‐free quantification was performed using the intensity‐based absolute quantification (iBAQ) algorithm embedded in MaxQuant to relatively quantify the proteins discovered.^[^ ^31^, ^52^ ^]^ Parameter for FDR was set to 1%. A minimum of one razor or one unique peptide was required for protein identification. Post‐translational modification (PTM) settings allowed for carbamidomethyl modification on cysteine residues and variable oxidation on methyl residues and acetylation of the N‐terminus, with 5 maximum modifications per peptide. Instrument settings were set to Orbitrap machine settings and were kept standard. Digestion mode was set to specific, using Trypsin/P and allowing for 2 missed cleavages maximum. The database search to look for peptide and protein sequences was performed using the reviewed human SwissProt database (UP000005640, downloaded on 17 March 2022), including the standard contaminants included with the MaxQuant software version 2.1.0.0. The 2160 identified proteins and obtained iBAQ values were anlaysed with the Perseus software (version 2.0.3.1). First, proteins only identified by site, reverse, and potential contaminants, in that order, were removed from the matrix leaving 2068 identified proteins. GOCC name and GOCC slim names were annotated using the majority protein IDs column and the mainAnnot.homo_sapiens.txt gz database comprising the human proteome downloaded using the annotation download tool in Perseus on 17 March 2022. iBAQ values were log2(x) transformed. Plasma membrane‐associated proteins were defined using GOCC terms “plasma membrane” and “plasma membrane part”. Any protein that has the GOCC annotation, “mitochondrial” or “mitochondrion” were considered to be mitochondrial. Proteins of the cytoskeleton were defined by GOCC terms “Cytoskeleton” and “Cytoskeleton part.”. Golgi and ER were defined as any GOCC terms encompassing “endoplasmatic reticulum” and “Golgi”. For the nucleus, “nuclear envelop”, “nuclear membrane”, “nuclear membrane part”, “nuclear pore”, “nucleolus”, “nucleoplasm” “nucleoplasm part” and “nucleus” were used. For endosomes and vesicles, GOCC terms encompassing “endosome”, “phagosome”, “endocytic vesicle”, “endomembrane system”, “endosomal part” and “vesicle” were considered. The fraction or number of proteins belonging to the different compartments was investigated in the following way: the compartments were removed in order according to the just‐defined GOCC terms. Then, using the “summarize columns” function in Perseus, the number and quantity of the remaining proteins after filtering were computed. The number of membrane‐associated proteins were counted per sample and their respective percentages were calculated using Excel. To calculate the % of total iBAQ, log2(x) transformed values were 2^x^ transformed and not a number (NaN) values were replaced with 0. The fraction of total iBAQ (% iBAQ) was then determined as follows:

(1) $$\begin{array}{ccc} & & \%\;\mathrm{Total}\;\mathrm{iBAQ}\;\mathrm{Membrane}\;\mathrm{associated}\;=\\ & & \left(\frac{\sum total\;iBAQ\;per\;sample}{\sum iBAQ\;of\;GOCC\;terms\;associated\;with\;membrane\;per\;sample}\right)x100\% \end{array}$$

To calculate the fold changes, first, each sample was normalized by subtracting the most frequent value per column from the iBAQ values in order to accommodate for the difference in the amount of sample injected into the mass spectrometer. Fold changes in iBAQ against the homogenate were calculated as 2^(iBAQpurified‐iBAQhomogenate)^ and against the abundance in the crude sample as 2^(iBAQpurified‐iBAQcrude)^.

The percentage of total iBAQ per protein in the purified sample was determined as follows:

(2) $$\%\;total\;iBAQ=\frac{2^{\mathrm{iBAQvalue}}}{\sum total\;iBAQ\;purified\;per\;sample}$$

The 20 most abundant plasma‐associated proteins, the highest fold changes compared to the abundance in the crude sample, the most abundant proteins not found in the homogenate, and the proteins evaluated in the western blot were determined. Their location was investigated by manually searching on https://www.proteinatlas.org/. The protein location was subdivided into plasma membrane (green), cytoplasm (black), vesicles, ER, nucleus (blue), cytoskeleton (yellow), and mitochondria (orange). Data was plotted in GraphPad Prism (version 8.4.3 (686)).

### Preparation of Liposomes and CML Using Thin Layer Evaporation and Extrusion

For the preparation of liposomes and CMLs, the thin layer evaporation and extruding method was used. The lipids were bought from Avanti Polar Lipids (Alabaster, Alabama, United States). The lipids used in the work are 1,2‐dioleoyl‐sn‐glycero‐3‐phosphocholine (DOPC 18:1 PC (cis)), 1,2‐dioleoyl‐sn‐glycero‐3‐phosphoethanolamine (DOPE, 18:1 PE), 1,2‐dioleoyl‐sn‐glycero‐3‐phospho‐(1′‐rac‐glycerol) sodium salt (DOPG, 18:1 (Δ9‐Cis) PG), cholesterol and 1,1′‐Dioctadecyl‐3,3,3′,3′‐Tetramethylindocarbocyanine Perchlorate (DiI, Sigma–Aldrich, The Netherlands) as fluorescent lipid. The lipids were dissolved in CHCl_3_ (Sigma–Aldrich, The Netherlands), aliquoted at 10 mg mL^−1^, and stored at −20 C°. The appropriate mass of lipids was placed in glass vials, mixed, dried with N_2,_ and left under vacuum overnight. The obtained lipid film was redissolved in PBS that was filtered (Whatman FP30/0.2, 0.2 µm filters) with 0.05% Sodium Azide (Sigma–Aldrich, The Netherlands) with or without added K562 plasma membrane extract in ratio 1:10 protein:synthetic lipid (wt/wt). The lipid film was slowly agitated for 2–3 h at room temperature. When the lipid film was completely dispersed, the sample was freeze‐thawed 8 times in liquid nitrogen and in a water bath at room temperature. Afterward, the sample was passed 10 times through a 0.4 µm polycarbonate filter (Avanti Polar Lipids), followed by extrusion 21 times through a 0.2 µm filter (Avanti Polar Lipids), using an Avanti Mini‐Extruder (Avanti Polar Lipids). The samples were stored at 4 C° and used for up to 4 weeks.

### Lipid Quantification After Extrusion

A fluorescence assay was used to determine the concentration of lipids after extrusion based on the fluorescent lipid DiI to account for the lost lipids during the procedure. For each lipid composition, a calibration curve with known concentration of lipids was made by taking a sample of the lipid mixture after freeze‐thaw, but before extrusion. The total lipid concentration was estimated using the DiI fluorescence as readout. The extruded samples were diluted 4 times with PBS before adding to the 96‐well plate (Greiner black). The calibration standard was made from 0.5 to 0.0625 mg mL^−1^ since higher concentrations showed diminishing fluorescence. Fluorescence was measured at room temperature with excitation λ = 485 nm and emission λ = 555 nm with cutoff λ = 550 nm in a SpectraMax Gemini XPS microplate spectrofluorometer instrument. The concentration was calculated afterward using the calibration curve.

### Size Distribution by DLS and Zeta Potential Measurements

The hydrodynamic diameter and size distribution of the CMLs and the liposomes were determined by dynamic light scattering (DLS), using a Malvern Zetasizer Nano ZS, which was also used for zeta potential measurement. The samples were diluted to ≈25 µg mL^−1^ lipids in PBS or dH_2_O, or to 100 µg mL^−1^ in a complete cell culture medium with 10% FBS. Measurements were performed at 25 C° using disposable folded capillary cells (Malvern Instruments Ltd., Worcestershire, UK). Each measurement was repeated 3 times and the average of a triplicate measurement is shown in figures.

### Small‐Angle X‐Ray Scattering

SAXS experiments were performed at the Multipurpose X‐ray Instrument for Nanostructural Characterization (MINA) at the University of Groningen. The instrument is equipped with a high‐intensity Cu rotating anode X‐ray source, providing a parallel collimated X‐ray beam with a photon wavelength of λ = 0.1543 nm. In order to explore a broad q‐range (0.1–8 nm^−1^), the SAXS data were acquired using two different sample‐to‐detector distances of 1.17 and 0.28 m. The scattering patterns were collected using a 2D Bruker Vantec500 detector (number of pixels 1024 × 1024; pixel size 136 µm x 136 µm). The samples were prepared by loading the liposome samples in a sealed glass capillary of 1.5 mm outer diameter with 0.01 mm wall thickness and then measured under vacuum. The SAXS patterns were converted into the 1D scattering intensity profiles using Fit2D software. After subtracting the scattering signal from the solvent background, the two data sets were merged to generate the final SAXS curves, where the scattering intensity profiles are plotted as a function of the modulus of the scattering vector q = 4πsinθ/λ. The sample‐to‐detector distance and the beam center position were calibrated using the scattered rings from a standard silver behenate powder sample.

The SAXS data was fitted using a Bilayer Gaussian model. The software package SASfit was used to accomplish the fitting.^[^ ^53^ ^]^

### Atomic Force Microscopy

AFM experiments were performed in liquid in Quantitative Imaging (QI) mode using the JPK Nanowizard ultra‐speed AFM. To immobilize the nanoparticles, poly‐L‐lysine coated glass was prepared freshly prior to the experiments following a published protocol.^[^ ^47^ ^]^ Briefly, coverslips were cleaned using EtOH, HCl (97%/3% v/v) and incubated with a 0.01 mg mL^−1^ solution of poly‐L‐lysine for 1 h after which the coated glass was washed with dH_2_O and dried in a 37 °C stove. The stock nanoparticle solution was diluted 1:60 with PBS and 50 µL of the sample was loaded on top of the previously poly‐L‐lysine coated glass (≈0.015 mg mL^−1^ lipids in 50 µL). Later, the chamber was filled up with PBS. Measurements were performed using qp‐BioAC CB3 probes (Nanosensors) generally having spring constants between 0.03 and 0.09 N m^−1^. Before imaging, cantilevers were calibrated using a contact‐base and thermal noise method included in JPK Nanowizard control (version 6) before each session. The QI images obtained during the experiment with increasing force (steps of 10 pN starting at 80 pN up till 170 pN) were recorded in such a way that all the other imaging parameters were kept constant (128 × 128 pixels, 3 µm x 3 µm, 15 ms pixel time, 300 nm lift‐height). Liposomes were imaged 11 days after preparation and CML particles after 14 days. All images were processed using the JPK Data processing software (version 6.1)

Only particles that were visible at 80 pN, that were not laying on a bilayer, that could be observed as singular, cap‐like objects, and that during imaging stayed within full view, were included in the analysis. In rare cases (<5 particles), due to electronic or other noise, a particle was not observed for 1 to 2 images but was clearly visible at the next image(s). In that case, only for those images where the height could be clearly determined, the height data was incorporated in the normalized height data. For the height plots in the main text, only particles that survived three consecutive pressures were included, and for the survival plots all eligible particles, excluding multilamellar particles, and according to previously stated constraints, were tracked and their heights measured in the JPK software.

### Uptake Studies by Flow Cytometry

The uptake of the CMLs and liposomes by cells was measured using flow cytometry. The MS5 cells were seeded in complete medium (cell culture medium supplemented with 10% FBS) in 24 well plates (Greiner) at a seeding density of 30000 cells/well. After 24 h, the medium was replaced with the nanoparticle dispersion at a concentration of 10 µg mL^−1^ lipids in medium with 10% FBS. Cells were exposed to nanoparticles at different times. After exposure, to wash away the extracellular nanoparticles and reduce the nanoparticles that are adhering to the cell membrane, the cells were washed 1 time with 1 mL medium with 10% FBS, followed by 2 washes with 500 µL PBS. After washing, the cells were harvested using 250 µL trypsin‐EDTA in PBS (TEP) and incubated for 5 min at 37 °C. The TEP was inactivated with 1 mL medium with 10% FBS, and the cells were collected by centrifuging the sample at 300 g for 5 min. The cells were resuspended in 100 µL PBS for measurements. For uptake studies in the K562 cell line, cells were seeded in a medium with 10% FBS in 24 wells plates (Greiner) at a seeding density of 50.000 cells/well. After 24 h, 35 µg mL^−1^ nanoparticles in 100 µL complete media were added on top of the old media, resulting in a volume of 350 µL/well and a concentration of 10 µg mL^−1^, and mixed by shaking the plate back and forward. Uptake of the nanoparticles in the K562 cell line was measured at different exposure times. At each time point the washing steps were the same as described for MS5 cells, but after each washing step the cells were collected by centrifuging at 300 g for 5 min. Cell fluorescence was measured using a Cytoflex S flow cytometer (Beckman Coulter).

### Adhesion Studies by Flow Cytometry

For adhesion experiments, MS5 cells were seeded at a density of 50000 cells/well in a 24 wells plate. 24 h after seeding, the plates were cooled to 4 °C for 1 h. Afterward, the cells were placed at 4 °C in a cold room and 10 µg mL^−1^ of nanoparticles were added to pre‐cooled medium and then to the cells. Adhesion was measured at different exposure times. At each time point, the medium with particles was discarded and the cells were washed once gently with 500 µL PBS. As a next step, 250 µL new medium was added and the cells were incubated at 37 °C for at least 3 h, in order to allow the nanoparticles adhering to the surface of the cells to be internalized. Finally, the cells were washed and prepared for flow cytometry as described above.

### Processing the Flow Cytometry Data

Flowjo software (version 10.8.1) was used to analyze the results of the flow cytometry. The forward scattering (FSC) was plotted against the side scattering (SSC), and gates were set to exclude the debris from the cell population. In the double scatter plot of FSC‐H (height) versus FSC‐A (area), gates were set to exclude the cell doublets. For each sample, the fluorescence of 20000 cells was measured and for each condition, 3 replicate samples were made. For each condition, the average median cell fluorescence of the 3 replicate samples was calculated and the results are shown in graphs made in GraphPad Prism (version 8.4.3 (686)).

### Sodium Azide Experiment

To deplete cell energy, cells were incubated with medium with 10% FBS supplemented with sodium azide (NaN_3_) (6688, Merck) at a concentration of 5 mg mL^−1^ for 30 min. Afterward, nanoparticles were exposed to the cells in medium with 10% FBS and NaN_3._ An identical experiment was performed in parallel, where cells were incubated with the nanoparticles in standard conditions, without the added NaN_3_ and preincubation. Harvesting was performed as described above. Experiments were performed with three replicate samples for each condition.

### Confocal Imaging of Nanoparticle Uptake in Live Cells

For confocal imaging on MS5 cells, 30000 cells/well were seeded in 35 mm dishes with a 170 µm thick glass bottom. 24 h after seeding, the cells were washed 3 times with serum‐free medium and incubated with 10 µg mL^−1^ liposomes and CMLs in medium supplemented with 10% FBS for 3 h and 24 h at 37 °C. At each time point, the cells were first washed with a medium supplemented with 10% FBS 3 times, and then with PBS 3 times. To visualize the lysosomes, cells were incubated with 100 nm Lysotracker Green (Thermo Fisher Scientific) in medium supplemented with 10% FBS for 30 min at 37 °C. After this step, the cells were washed again three times with serum‐free medium and three times with PBS. To stain the nuclei 1µg mL^−1^ Hoechst 33342 solution (Thermo Fisher Scientific) in complete medium was used and incubated for 5–10 min at 37 °C. Finally, the cells were washed with PBS once and incubated in a complete medium for imaging. In total, images were taken roughly 45 min after stopping exposure. During and after the staining steps, the Petri dish was packed in aluminum foil to protect fluorophores from light. The live cells were imaged with the Zeiss Cell Discoverer 7. DiI was excited with a 524 nm laser, LysoTracker G stainedreen with a 427 nm laser, and Hoechst with a 411 nm. The ImageJ software was used for adjusting the brightness and contrast of the lysosome channel to allow better visualization.

### Statistical Analysis

Figure 1 and Table 1 show the average and standard deviation over 5 replicate extractions of the amount of proteins recovered from K562 in the different fractions (mean ± SD, *n* = 5). Similar values were obtained for MS5 cells. The western blots shown in the manuscript were repeated multiple times with similar results (except for Figure S3, Supporting Information, which was done once). The experiment in Figure 4c–g was performed once with 3 replicate samples for each condition, with one batch of particles, The results in Figure 4h (HeLa cells) were repeated multiple times with different batches in triplicate, and similar results were obtained in each experiment (Figure 4h shows one example). The data shown is the averaged median fluorescence intensity and standard deviation (SD) over the 3 replicate samples (mean ± SD, *n* = 3). One‐way analysis of variance (ANOVA) with Tukey correction was used to compare the uptake of liposomes and CMLs after 6 h exposure (*n* = 3). P values are reported and p < 0.05 was considered statistically significant. Figure 5a shows 3 repeated measurements of the size distribution of plasma membrane extracts (*n* = 3). Figure 5b–e, shows the average and SD of the results obtained in 7 independent experiments, performed with various extracts and batches of nanoparticles, each in triplicate (mean ± SD, *n* = 7). SAXS measurements in Figure 5 were performed with one batch of particles. The AFM was repeated with multiple batches of particles giving similar results (Figure 6 shows one example). Experiments in Figure 8 were repeated 4 or 5 times in triplicate (*n* = 4 or 5). The average and SD over 3 replicate samples are shown for each individual experiment (mean ± SD, *n* = 3). In addition, the results obtained for CMLs were normalized by those obtained for liposomes, and for the normalized results, the average and SD over the replicate experiments are also shown (mean ± SD, *n* = 4 or 5).

All graphs and statistical analysis were performed with GraphPad Prism (version 8.4.3 (686)).

## Conflict of Interest

The authors declare no conflict of interest.

## Supporting information



Supporting Information

## Data Availability

The data that support the findings of this study are available from the corresponding author upon reasonable request.
