# Supplementary material for: Optimization of Cell Membrane Purification for the Preparation and Characterization of Cell Membrane Liposomes
Source: Small Methods. 2024 Oct 21;8(12):2400498. doi: 10.1002/smtd.202400498 (PMC11671854; doi:10.1002/smtd.202400498)
Supplement: Supplementary file 1 — Supporting Information [file SMTD-8-2400498-s001.docx]

Supporting Information

Optimization of Cell Membrane Purification for the Preparation and Characterization of Cell Membrane Liposomes.

Sander de Weerd, Emma A Ruiter, Eleonora Calicchia, Giuseppe Portale, Jan Jacob Schuringa*, Wouter H. Roos*, Anna Salvati*

S. de Weerd, E.A. Ruiter, E. Calicchia, A. Salvati

Nanomedicine and Drug Targeting

Groningen Research Institute of Pharmacy, University of Groningen, A. Deusinglaan 1, Groningen, 9713 AV, The Netherlands

E-mail: a.salvati@rug.nl

E. Calicchia, G. Portale

Macromolecular Chemistry & New Polymer Materials

Zernike Institute, University of Groningen, Nijenborgh 4, 9747 AG, The Netherlands

S. de Weerd, W.H. Roos

Molecular Biophysics

Zernike Institute, University of Groningen, Groningen, Nijenborgh 4, 9747 AG, The Netherlands

S. de Weerd, J.J. Schuringa

Department of Experimental Hematology

University Medical Center Groningen, University of Groningen, Groningen, 9700 RB, The Netherlands

**Supporting Protocol S1 – Protocol for plasma membrane isolation using nitrogen cavitation. Modified from Suski *et al.***^[17]^

**1.0 Materials:**

**EGTA 100 mM =** for 50 mL: 1,901 g EGTA + 50 mL MQ, add NaOH 1M to adjust pH to 8, when it dissolves.

**MgCl_2_ 100 mM.**

**Starting Buffer (SB):** 20,5g D-Mannitol, 13 g Sucrose, 400 mL Milli-Q (or dH2O), 1,8171 g Tris-Base. Place the buffer at 4 °C to cool (preferably overnight), adjust the pH to 7,4 with concentrated HCl, adjust volume to 500 mL with Milli-Q or dH_2_O. The buffer can be stored for 2 weeks in a glass bottle at 4 °C.

**Plasma Membrane Resuspension Buffer (PMRB):** 0,523g Bis-tris in 400 mL dH_2_O. Add 1 mL of 100 mM EDTA. Place at 4 °C (preferably overnight), adjust the pH to 6,0 with HCl and adjust volume to 500 mL. The buffer can be stored for 2 weeks in a bottle at 4 °C. (EDTA can become inactive in glass, be wary)

**2.0 Preparation:**

Place ultracentrifuge rotors SW32-Ti (Beckman & Coulter) and SW41-Ti (Beckman & Coulter) in the cold chamber including the buckets. Place the nitrogen cavitation vessel (Parr instruments or equivalent) in 4 °C.

**2.1 Prepare gradient solutions:**

On the day of the experiment, prepare 38, 43 and 53 % (wt/wt) sucrose solutions in **PMRB**.

38%: 19 g + 31 g **PMRB**

43%: 21,5 g + 28,5 g **PMRB**

53%: 26,5 g + 23,5 g **PMRB**

Place these buffers in Falcon tubes at 4 °C while agitating to dissolve.

**2.2 Prepare fresh buffers from SB:**

**Storage Buffer (StB):** Dissolve the Roche cOmplete™, EDTA-free Protease Inhibitor Cocktail tablet first in SB (1 tablet is meant for 50 mL), allow for the tablet to dissolve completely, then, take 2 mL and place in a tube at 4 °C. This will be the Storage buffer (**StB**).

**Isolation buffer (IB):** 50 mL of **SB** + 1 c0mplete mini Roche EDTA free protease inhibitor cocktail, + 0,5 mM EGTA (using 100mM EGTA solution) + 2,5 mM MgCL_2_ (using MgCl_2_ 100 mM solution). Magnesium should keep internal membranes intact. DNAse (10 µg mL^-1^), RNAse (10 µg mL^-1^) and benzonase (1 unit per 35 mL) were added for the extraction from MS5 cells.

**Keep buffers on ice and start cooling empty Falcon tubes required for the collection of the cells**

*Note: make sure to use the appropriate buffer during the protocol (****SB****,* ***StB****,* ***IB****,* ***PMRB****).*

**3.0 Method:**

**All centrifugations are performed at 4 °C.**

For K562 use approximately 0,5 billion cells. For adherent cells use a cell scraper or a cell scraper in combination with 2 mM EDTA in PBS (optimize incubation time and concentration per cell) to detach the cells prior to lysis. Avoid using trypsin. Lysis pressure should be evaluated on a per-cell basis and is depending on the use of EDTA for harvesting.

1. Collect cells in 50 mL Falcon tubes by placing 50 mL of medium or PBS with cells in them and spinning down at 300 g for 5 minutes at 4 °C.
2. Afterwards, rinse the flasks once more with PBS, which can be re-used later for the washing steps.
3. After all the cells are in two tubes, start washing the cells twice with cold PBS and centrifuge at 300 g for 5 minutes. For this re-use the PBS that has been used to wash the flasks that contained the cells.
4. The last time, place the cells in 1 Falcon tube, and wash one last time with **SB.** (300 g, 5 min.) Then, resuspend the cells in 35 mL of **IB**, or the appropriate volume for the size of the cell disruption vessel (nitrogen cavitation device) that is used**.** This will place the cells at approximately 10-15 million cells per mL of buffer.
5. Note: Here, dilute cells 20-50 x and count them in a hemocytometer.
6. Place the **IB** with the cells in the cell disruption vessel**.** Pressurizing the vessel to 300-320 psi (depending on cell-type) using the instructions in the booklet.
7. Let the sample equilibrate for 30 minutes. Shake the vessel to make sure the N_2_ goes into solution: if lots is absorbed (this can be observed as a pressure drop), readjust to 300-320 psi.
8. Decompress the vessel partly while collecting the **homogenate** in a 50 mL Falcon tube, do not do this too fast or the solution might splash out. After most of the pressure is lost, close all valves, place pressure in the nitrogen inlet hose, then, open the nitrogen filling inlet a little bit to re-pressurize the chamber to 300-320 psi, then, collect some more homogenate. Repeat until only N_2_ gas comes out. Generally, one re-pressurization is enough.
9. Note: Dilute the homogenate 10 x and count unbroken cells to determine the lysis efficiency.
10. Centrifuge 2 times at 800 g for 5 minutes discarding the pellet which contains nuclei, cell debris and unbroken cells. The supernatant contains the membrane fragments of interest, mitochondria, microsomes and other cytosolic proteins and other membranes.
11. Place the supernatant in the pre-chilled 38,5 mL Ultra Clear Ultra centrifuge tubes (Beckman Coulter, 38,5 mL, Open-Top Thinwall Ultra-Clear Tube, 25 x 89 mm) and use the SW32-Ti to centrifuge 2 x at 10.000 g for 10 minutes to pellet the mitochondria (Use deceleration: 3). Discard the pellet. Place the supernatant in a fresh 38,5 mL Ultra Clear centrifuge tube before spinning down a second time. Carefully balance the buckets.
12. After the last centrifugation at 10.000 g, the supernatant now contains membrane fragment vesicles and cytosolic proteins. Place the supernatant in pre-chilled 38,5 mL Ultra Clear Ultra Centrifuge tubes and centrifuge 1 x at 41.000 g for 25 minutes in the SW32-Ti to pellet the cell membrane fragments. (use acceleration 5, deceleration 5)
13. Remove the supernatant with an aspirator (sucking apparatus), being careful not to lose the pellet, but to remove all the liquid. Since the liquid consists of concentrated cytosolic proteins, be sure to remove as much as possible (with a tissue if needed). Resuspend the **pellet** in 35 mL **SB**, (the standard buffer without added magnesium, EGTA and inhibitors. This step is necessary to remove MgCl_2_ and cytosolic proteins before separation on sucrose). First resuspend the pellet in a small volume using, preferably, a few gentle strokes of a 2 mL Potter-Elvehjem homogenizer or, if not available, by pipetting, then adjust to 35 mL.
14. Centrifuge again at 41.000 g for 25 minutes. Remove the supernatant with the aspirator. (Use acceleration 5, deceleration 5)
15. In the meantime: **carefully** (by tilting the tube) layer the following sucrose gradient in two SW41 UltraClear centrifugation tubes (13,2 mL, Open-Top Thinwall Ultra-Clear Tube, 14 x 89 mm): 2,5 mL 53%, 3 mL 43%, 4 mL 38% (**other concentrations of sucrose might be required depending on the cell type**). Make two gradients and use the one that has the sharpest intersections, marking their position on the tube with a marker for reference. Use one of two gradients with the poorest intersections as balance in the next centrifugation or place the combined pellets from the 10.000 g step on the second gradient if a higher yield and less pure membrane fractions is also of interest.
16. **Note:** make the gradients fresh, as the intersections may fade over time.
17. Dissolve the pellet of the second 41.000 g centrifugation in 1,5-2 mL of ice cold **PMRB**. Be sure to properly resuspend using a couple strokes of the 2 mL Potter-Elvehjem homogenizer, as much as needed. **Carefully**, layer it on top of the sucrose gradient without disturbing the gradients’ interfaces. (Tilt the tube to do this)
18. Centrifuge for 2,5 hours at 95.000 g. (acceleration 5, deceleration 5) using a SW41-Ti Rotor.
19. A western blot for a membrane marker should be used to test all fractions and to determine where, for each specific cell type, the membrane fraction is. The fraction with the highest intensity membrane marker on the western blot is the membrane fraction. For K562 cells, with this protocol, the membrane fraction is in the top of the 38% sucrose layer and is shown as a thick white band. The white band interfacing 38% and 43% is mitochondrial contamination.
20. Poke a hole in the bottom of the ultracentrifuge tube using a (hot) needle.
21. Collect fractions in Eppendorf tubes (approximately 1 mL per fraction), trying to collect the different fractions in separate tubes with limited contamination.
22. Dilute the fraction with the plasma membrane band in 10 mL **SB** (Just SB, without magnesium EGTA and protease inhibitors)**.** Centrifuge for 1 hour at 95.000 g in 13,2 mL UltraClear centrifuge tubes to pellet the membrane fragment vesicles and to remove the sucrose.
23. Redissolve the pellet in 1-2 mL **StB**. For storage, make smaller aliquots (~200 µL) to limit the number of freeze-thaw cycles for the membrane extract. Store at – 80 °C. After defrosting, resuspend by pipetting up and down gently.

General remark: pellets can be tough to dissolve, use Potter-Elvehjem homogenizer and pipetting up and down at times. Be sure that the equipment is cooled to 4 °C.

**Supporting Table 1** – Table summarizing the different lipid compositions and membrane extracts used throughout the manuscript in the different Figures (main and supporting).

| **Figure number** | **Lipid Composition** | **Membrane** |
| --- | --- | --- |
| **Main text** | | |
| 4 | DOPC:DOPE:DOPG 2:1:1 with 10 mol% cholesterol, 0,5 mol% DiI | Liposome, Crude & Purified membrane |
| 5,6,7,8 | DOPC:DOPE:DOPG 2:1:1 with 10 mol% cholesterol, 0,5 mol% DiI | Liposome & Purified membrane |
| **Supporting Figures** | | |
| S5,S6,S7,S8,S9,S10,S11,  S12,S13,S14,S15 | DOPC:DOPE:DOPG 2:1:1 with  10 mol% cholesterol, 0,5 mol% DiI | Liposome & Purified membrane |
| S5 | DOPE:DOPC:DOPG 2:1:1 with  10 mol% cholesterol, 0,5 mol% DiI | Liposome & Purified membrane |
| S6,S7 | DOPC:DOPE:DOPG 2:1:1 with  33 mol% cholesterol, 0,5 mol% DiI | Liposome & Purified membrane |


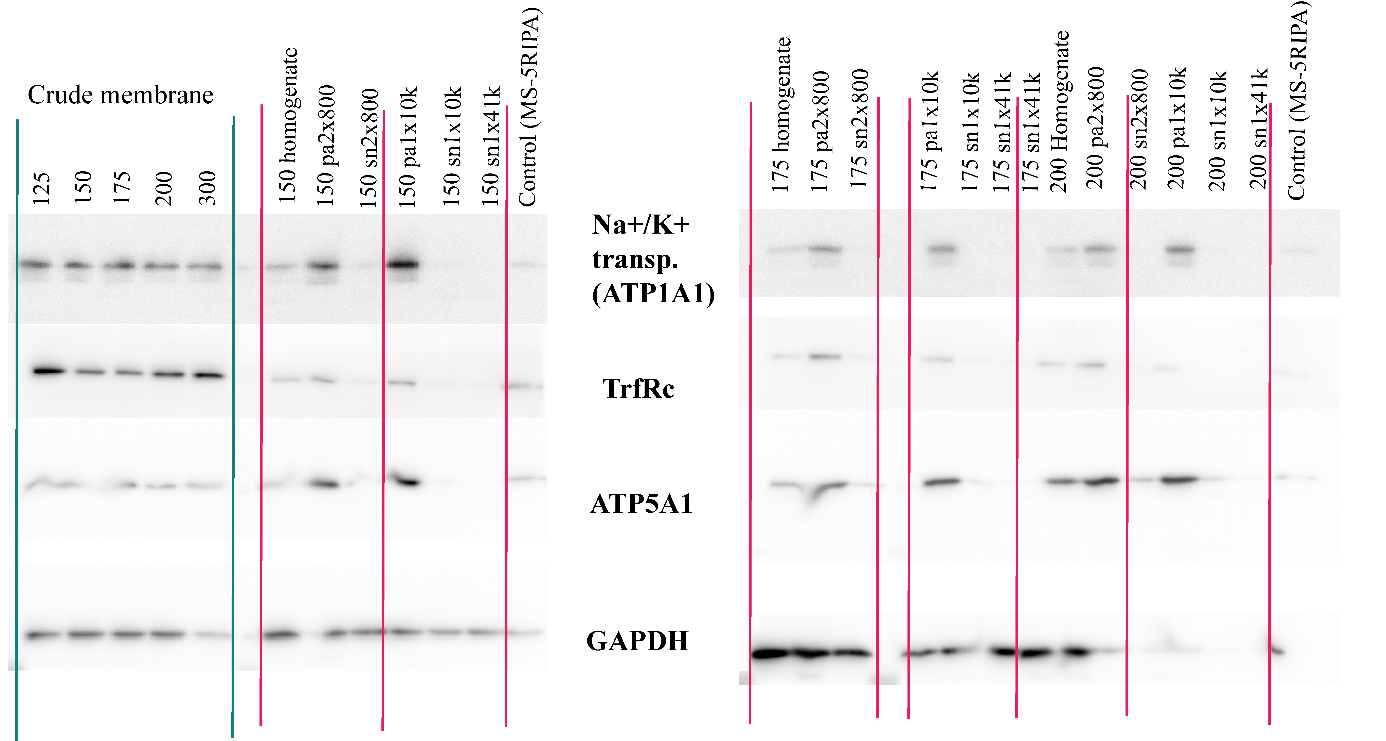


**Figure S1 –** Western blots of pellets, supernatants and final products obtained after lysis of MS5 cells using nitrogen cavitation at several pressures. The results for the purified cell membrane fraction is shown between the green bars. Controls are whole cell lysates generated using a standard RIPA lysis protocol. 10 µg of protein were loaded on each lane. Note that the pellet at 41.000 g was obtained after only one centrifugation, increasing the cytosolic content in the final product (green). Proteins were detected with antibodies against the plasma membrane ATPase Na^+^/K^+^ transporting subunit alpha1 (ATP1A1), transferrin receptor (TrfRc), mitochondrial membrane protein mitochondrial ATP synthase subunit a1 (ATP5A1), and cytosolic marker glyceraldehyde phosphatase (GAPDH). Using 10.000 g, mitochondria are removed from the supernatant, as evident by the bright spots at ATP5A1. Looking at the purified fractions, in green, the pressure did not seem to influence purity as much. Hence, based on the yield obtained, 200 psi was determined as the optimal pressure. (sn = Supernatant, pa = Pellet after)


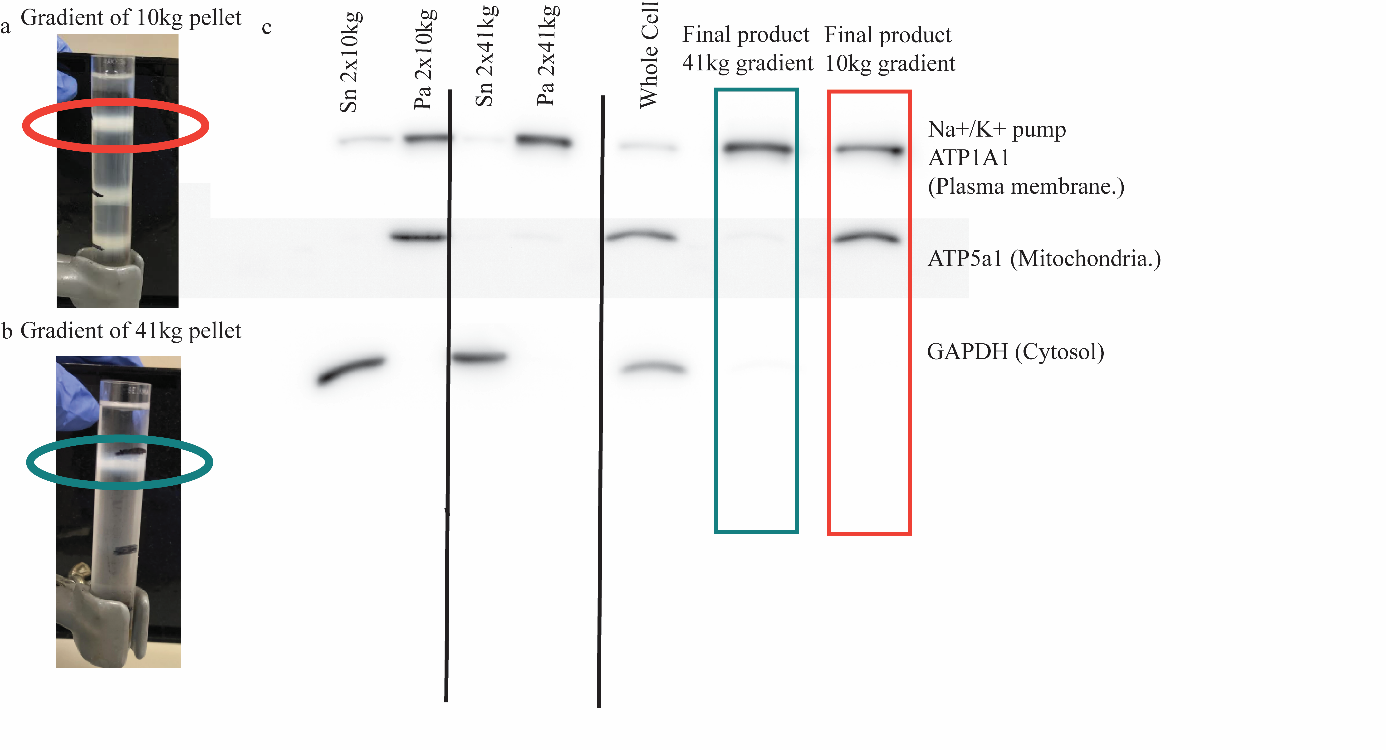


**Figure S2 –** Using MS5 cells, the membrane material obtained after centrifugation at 10.000 g and 41.000 g was layered on top of the sucrose density gradient and centrifuged at 95.000 g to obtain the separation seen in a) and b) respectively. c) Western blots of the fractions obtained in the described experiment from the two gradients. 10 µg of protein was loaded on each lane. The whole cell lysate (WCL) is a RIPA lysate of MS5 cells. Proteins were detected with antibodies against the plasma membrane ATPase Na^+^/K^+^ transporting subunit alpha1 (ATP1A1), mitochondrial membrane protein mitochondrial ATP synthase subunit a1 (ATP5A1), and cytosolic marker glyceraldehyde phosphatase (GAPDH). The results show that the product of the sucrose gradient fraction obtained after centrifugation at 41.000 g has almost no mitochondrial contamination, whereas mitochondrial contamination was observed when purifying the 10.000 g pellet on the sucrose gradient. Nevertheless, also in this case, the sucrose gradient is able to remove part of the contamination from mitochondrial membranes from the final product. Overall, these results show that the method can be easily transferred to adhering cells. (Pa = Pellet after, Sn = Supernatant) **
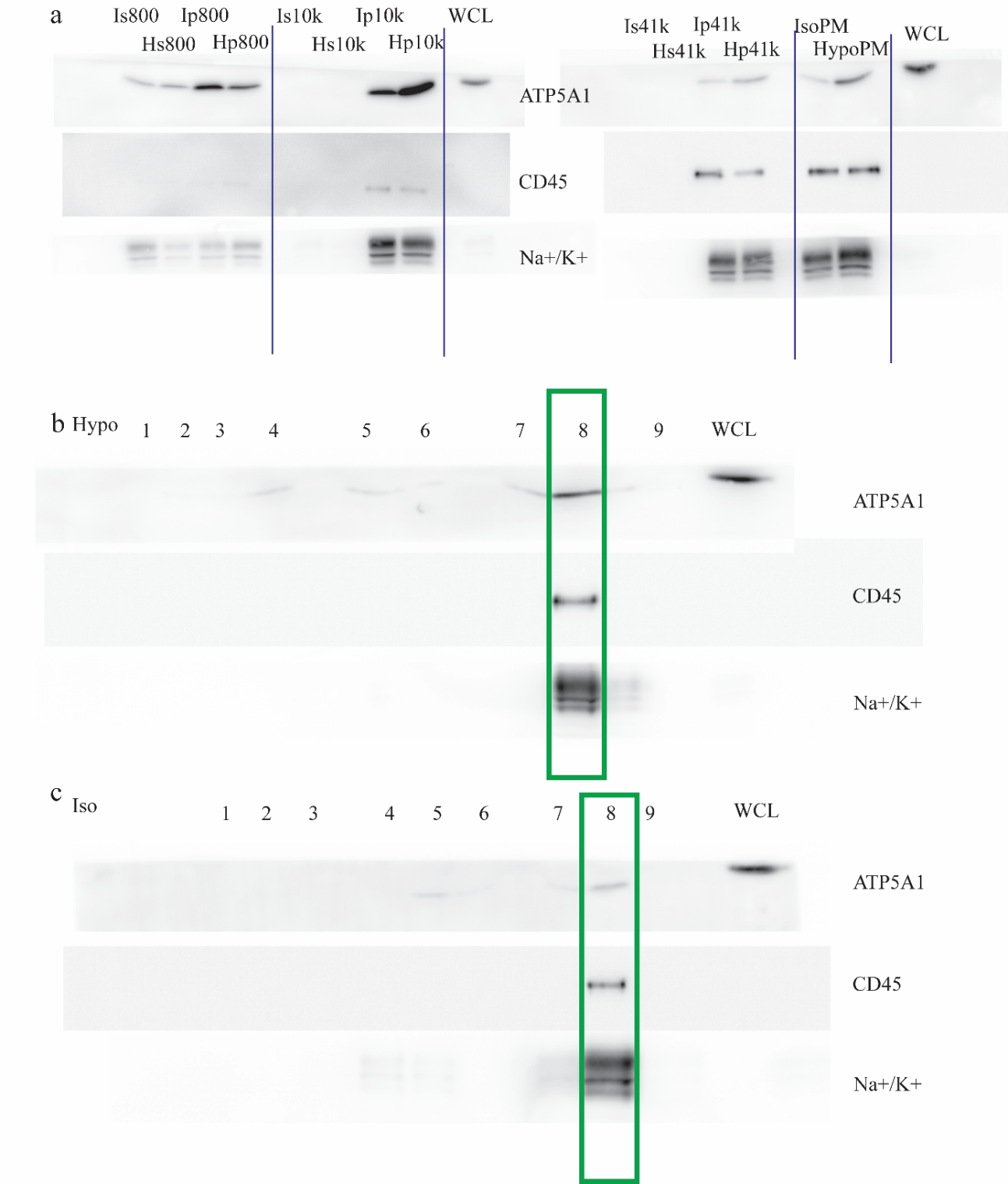
Figure S3.** Western blot analysis of the fractions recovered by sucrose gradient fractionation from the cell membrane extracted obtained by manual homogenization in isotonic and hypotonic buffers. Proteins were detected with antibodies against the plasma membrane ATPase Na^+^/K^+^ transporting subunit alpha1 (ATP1A1) andcluster of differentiation 45 (CD45) and the mitochondrial membrane protein ATP synthase subunit a1 (ATP5A1). 15 µg of protein was loaded on each lane. The whole cell lysate (WCL) control is a K562 RIPA lysate. a) Supernatants and pellets obtained using the two buffers, b) sucrose gradient fractions obtained after hypotonic lysis, c) sucrose gradient fractions obtained after isotonic lysis. Overall the results show that the differential centrifugation and sucrose gradient fractionation allow the extraction and purification of membrane fractions, with some contamination from mitochondria, in particular in hypotonic buffer. (Is = Isotonic Supernatant, Ip = Isotonic Pellet, Hs = Hypotonic Pellet, Hs = Hypotonic Supernatant, IsoPM is the plasma membrane obtained using isotonic buffer, HypoPM is the plasma membrane obtained using hypotonic buffer)


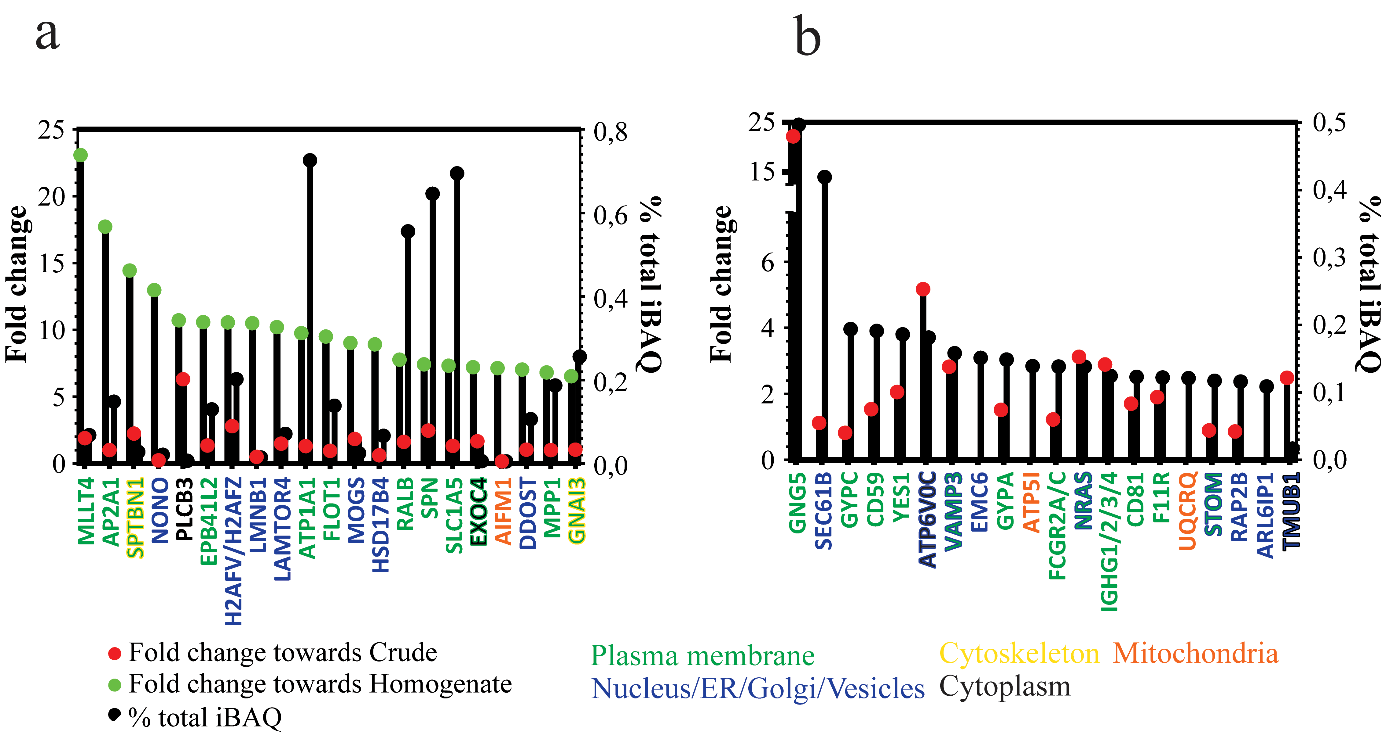


**Figure S4.** a) Proteomic results showing the proteins with highest fold change in respect to the homogenate, and b) for the proteins not found in the homogenate, the highest fold change in respect to the crude sample. Proteins that belong to more than one cellular compartment are colored with more than one color. The percentage in respect to the total iBAQ is also plotted.


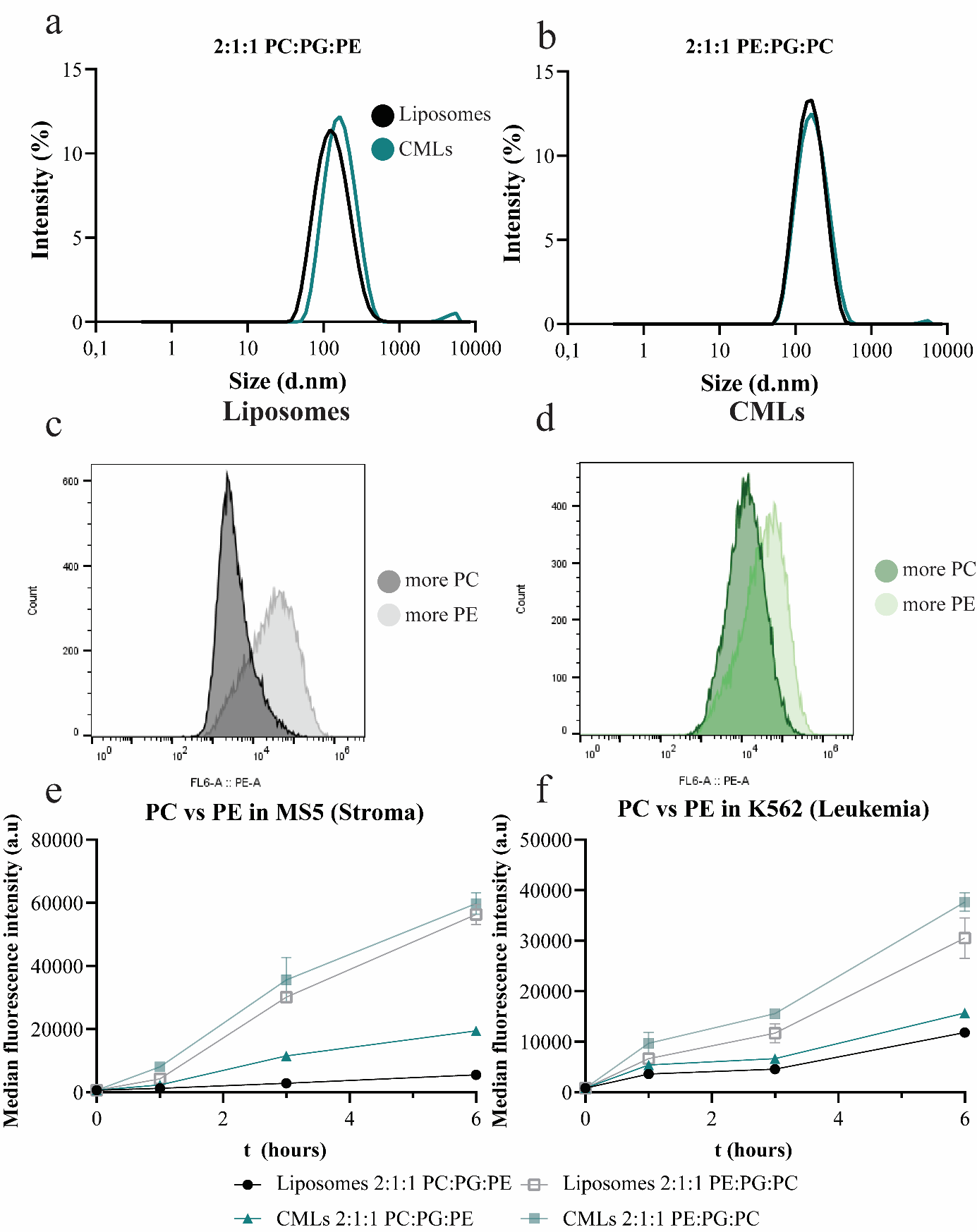


**Figure S5.** Comparison of liposomes and CMLs consisting of a mixture of 2:1:1 DOPC:DOPG:DOPE with 10 mol% cholesterol or of 2:1:1 DOPE:DOPG:DOPC with 10 mol% cholesterol. a, b) Size distribution obtained by DLS of (a) 2:1:1 PC:PG:PE particles in PBS (~25 µg mL^-1^). and (b) 2:1:1 PE:PG:PC particles. c, d) Cell fluorescence distribution of MS5 cells after 3 hours of exposure to 10 µg mL^-1^ (a) liposomes or (b) CMLs in 10% FBS. e-f) Fluorescence intensity of (f) MS5 cells and (f) K562 cells, exposed for increasing times to 10 µg mL^-1^ liposomes and CMLs in 10% FBS. The results are the average and standard deviation over 3 replicate samples of the median cell fluorescence intensity measured by flow cytometry (mean ± SD, n=3). The results obtained with the optimized liposomes and CMLs are shown again in Figure 8, where all experiment replicates are collected in order to show their high reproducibility for multiple membrane extracts and CML batches and in independent experiments (performed with cells at different passage number).

**
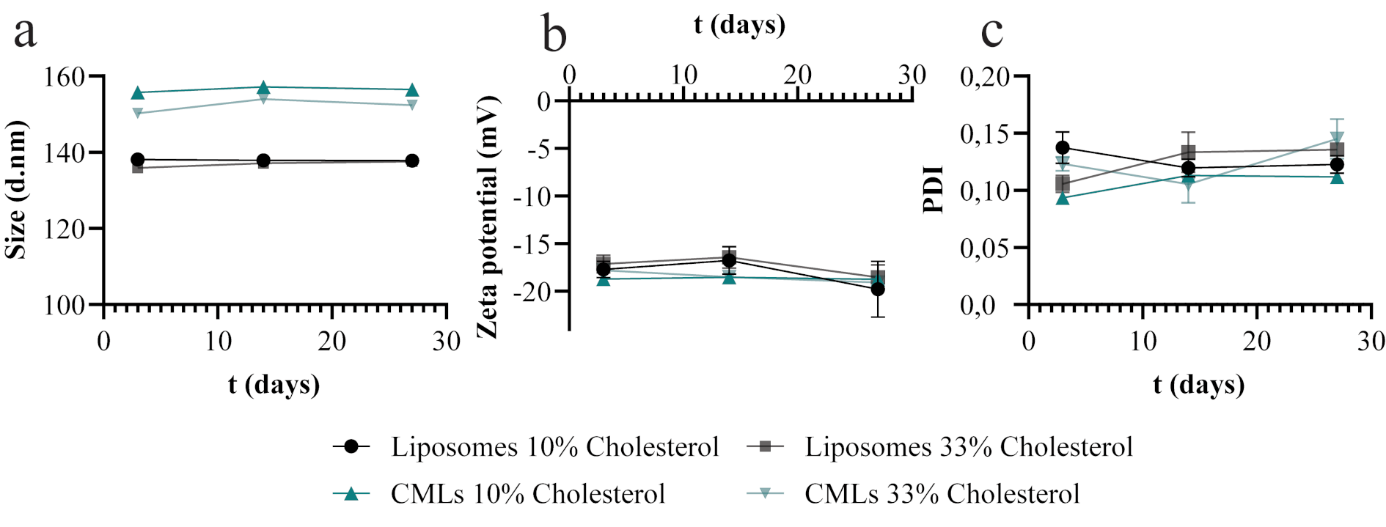
**

**Figure S6.** a) Size, b) PDI, c) and zeta potential of 33 and 10 mol% cholesterol liposomes and CMLs (~25 µg mL^-1^) measured 3, 14 and 27 days after preparation in PBS and storage at 4 °C. The results are the mean of a triplicate measurement, error bars depict the standard deviation (mean ± SD, n=3).

**
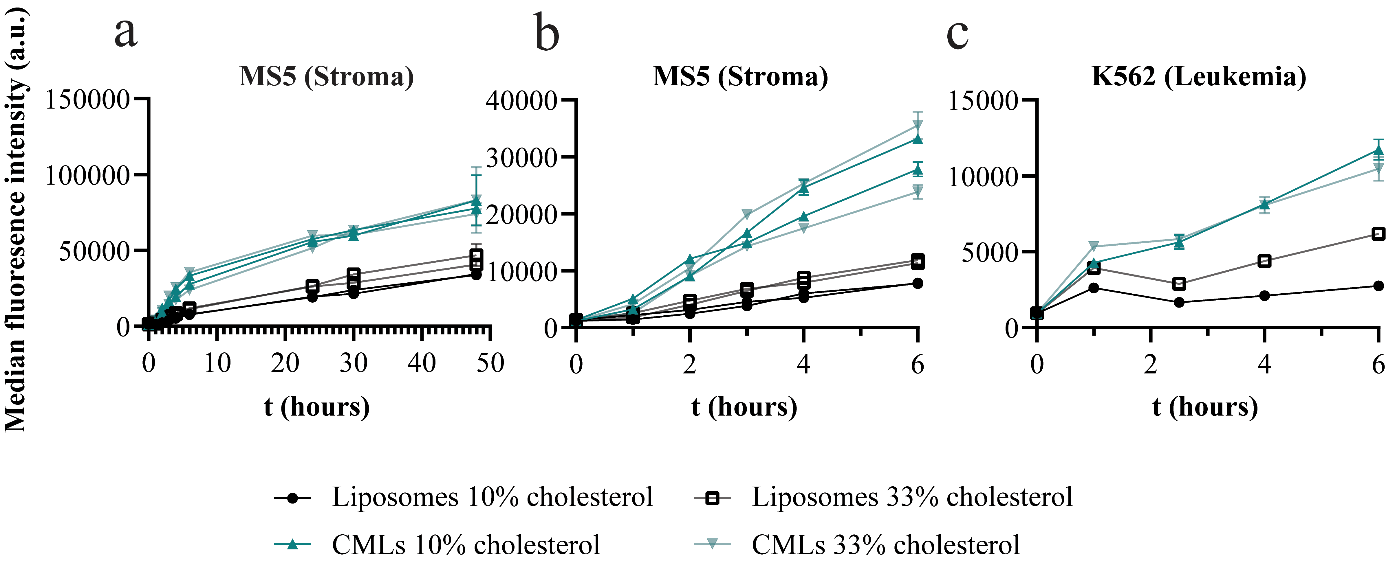
**

**Figure S7.** a) Median cell fluorescence of MS5 cells exposed to 10 µg mL^-1^ CMLs and liposomes in the presence of 10% FBS. b) The data of panel a are shown again for the first 6 hours of incubation. c) Median cell fluorescence of K562 cells exposed to 10 µg mL^-1^ CMLs and liposomes in the presence of 10% FBS. The results are the average and standard deviation over 3 replicate samples of the cell fluorescence intensity measured by flow cytometry (mean ± SD, n=3). The results obtained with the optimized liposomes and CMLs are shown again in Figure 8, where all experiment replicates are collected in order to show their high reproducibility for multiple membrane extracts and CML batches and in independent experiments (performed with cells at different passage number).


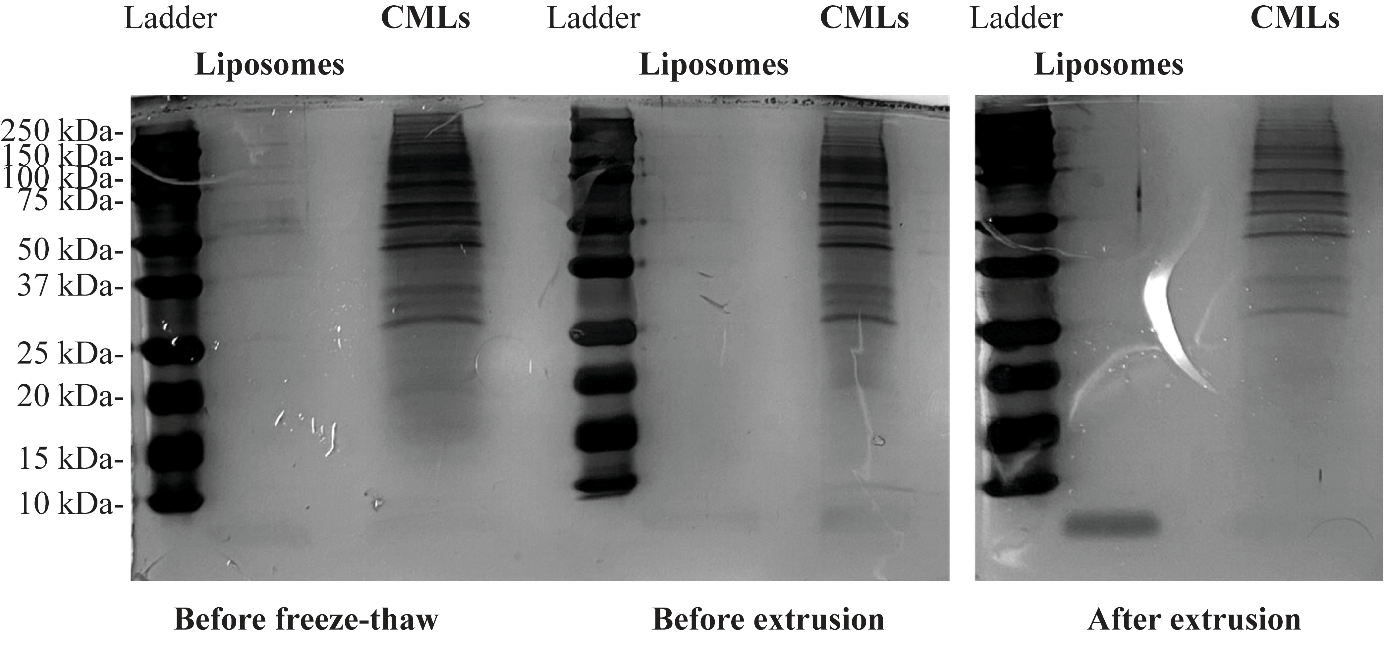


**Figure S8.** Silver staining of liposomes and CMLs before freeze-thawing, before extrusion, and after extrusion. Equal volumes were loaded on each lane.

**
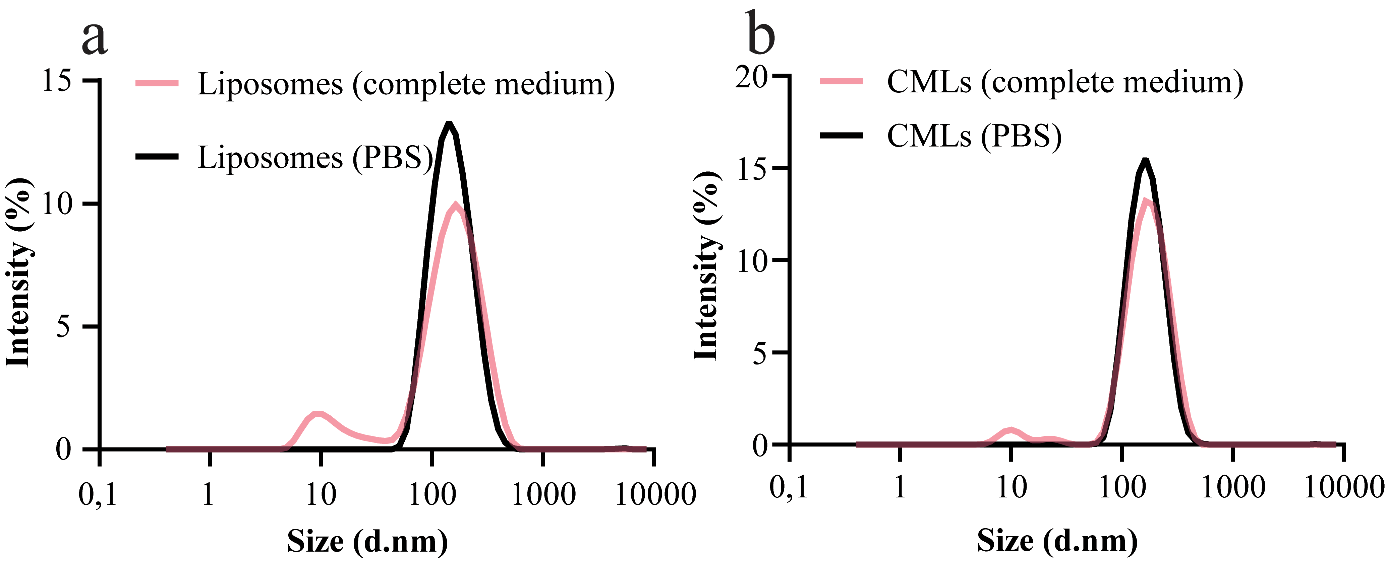
**

**Figure S9.** Size distribution of nanoparticles suspended in PBS and in media with 10% FBS. In the case of medium with FBS, an average of a triplicate measurement is shown, measured at 100 µg mL^-1^. The average size distribution of all batches of liposomes and CMLs in PBS is included in black for comparison.

**Table S2.** Table with parameters used for the fit of the SAXS results shown in Figure 5g (bilayer gaussian).

| **Parameters** | **Liposome** | **K562 cell membrane liposome** |
| --- | --- | --- |
| **σH** | 0,36 | 0,25 |
| **rhoH** | 1 | 1,15 |
| **σC** | 0,42 | 0,39 |
| **rhoC** | -1,97 | -2 |
| **zHH (nm)** | 3,6 | 3,68 |
| **N** | 1 x 10^-10^ | 1,68 x 10^-10^ |
| **bkg** | 2 x 10^-5^ | 1 x 10^-4^ |

**
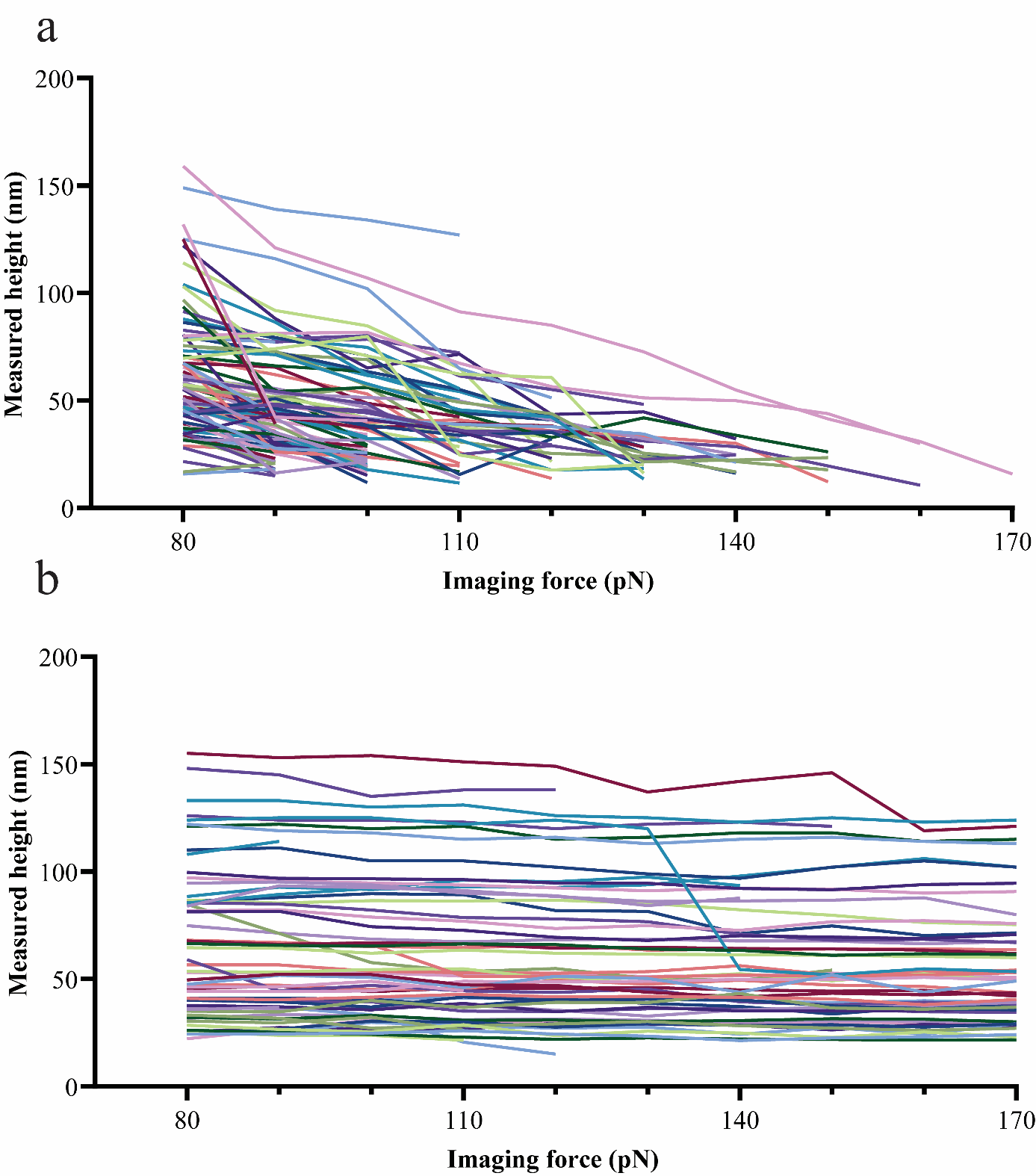
**

**Figure S10.** a-b) Heights obtained using AFM for individual (a) liposomes and (b) CMLs suspended in PBS and immobilized on poly-L-lysine coated glass as a function of increasing imaging force (pN).

**
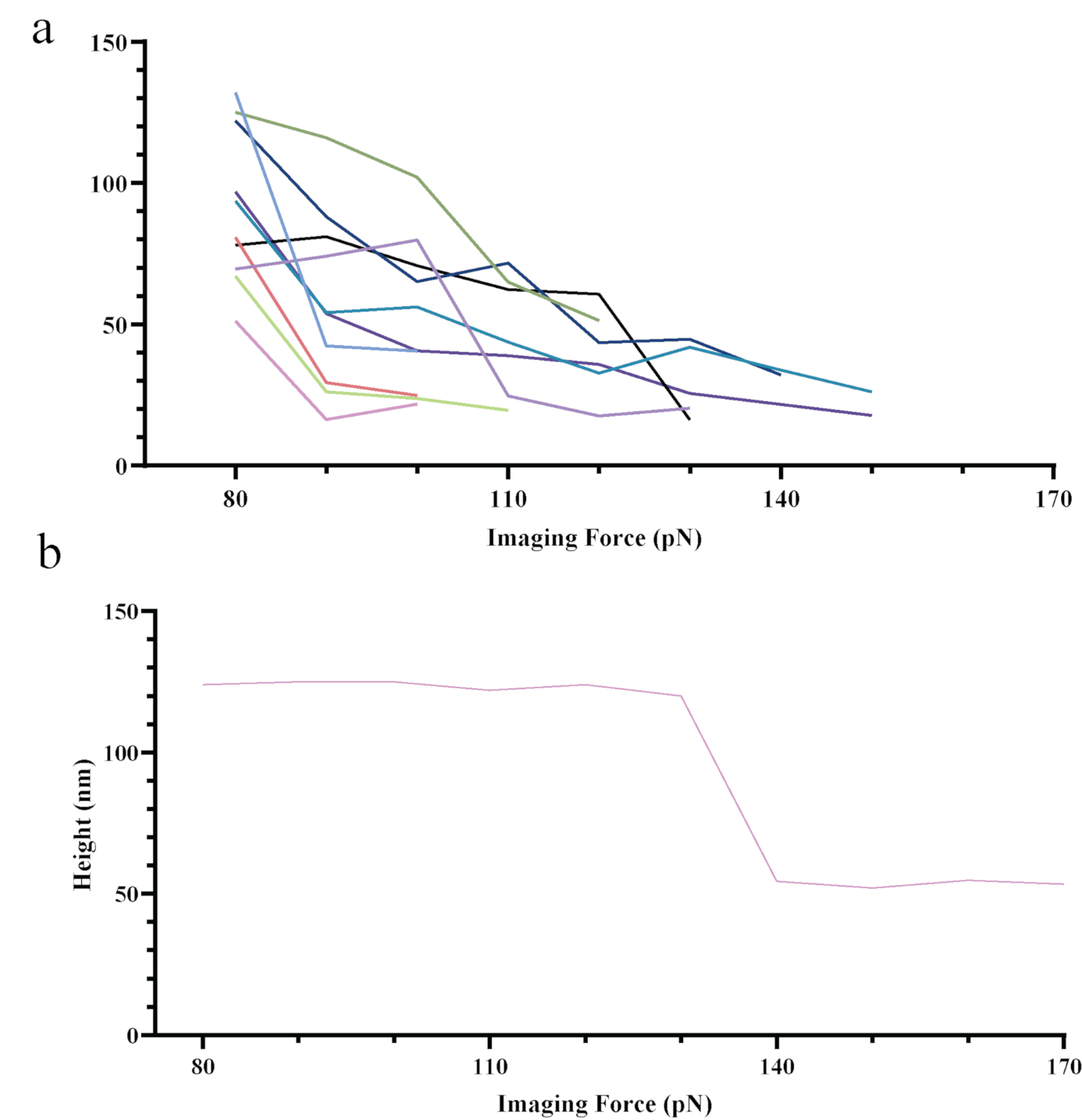
**

**Figure S11.** a-b) Height as a function of imaging force for (a) 10 liposomes and (b) 3 CMLs. The large drop in height (>30 nm) observed in 2 consecutive images suggested that these particles may be multilamellar. Because of this, these particles were excluded from the analysis (see main text for further explanations).

**
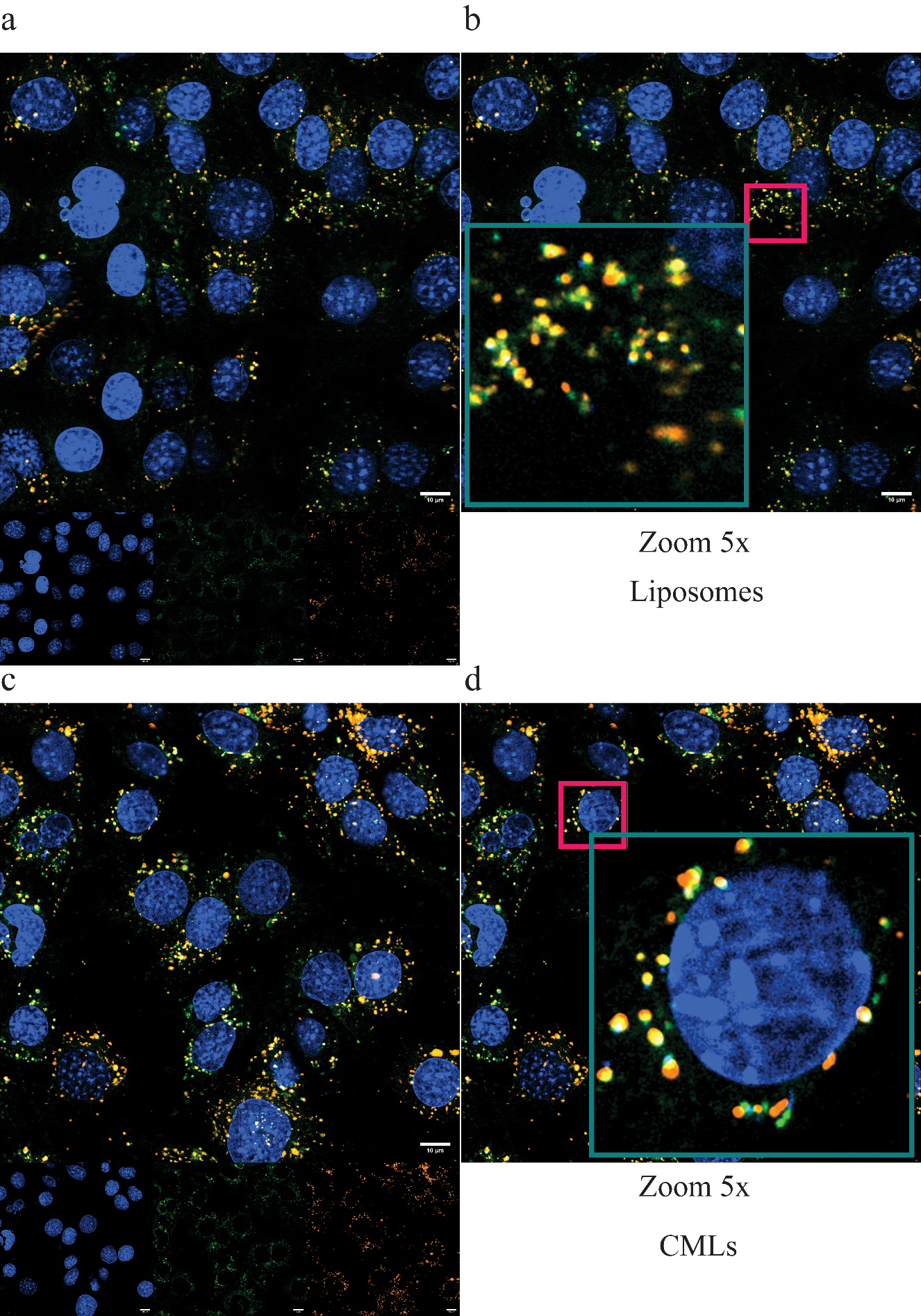
**

**Figure S12.** a) Live cell confocal microscopy image of MS5 cells exposed to 10 μg mL^-1^ (a, b) liposomes or (c, d) CMLs for 24 hours in 10% FBS. Blue = Hoechst stained nuclei, green = Lysotracker stained lysosomes, and red = DiI-labelled fluorescent nanoparticles. In the green inset in panels b) and d) the detail in the red square is shown after a 5x magnification. Scale bar: 10 µm. Brightness and contrast were optimized in ImageJ (Fiji). The results show that both CMLs and liposomes end up in the lysosomes.

**
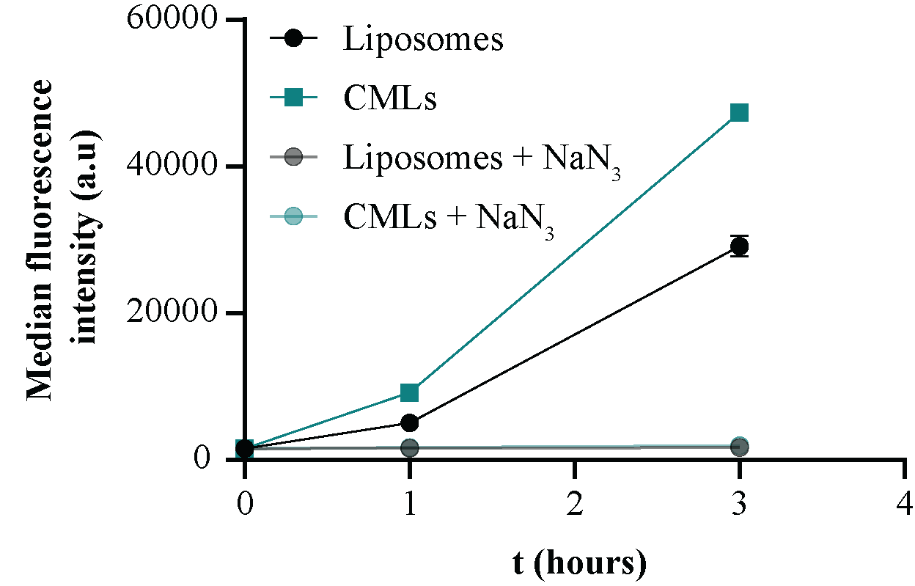
**

**Figure S13.** Fluorescence intensity of MS5 cells exposed for increasing times to 10 µg mL^-1^ liposomes and CMLs in 10% FBS in the presence of 5 mg mL^-1^ NaN_3_. Part of the cells were exposed to the liposomes and cell membrane liposomes in standard conditions and part in the presence of NaN_3_. The results are the average and standard deviation over 3 replicate samples of the median cell fluorescence intensity measured by flow cytometry (mean ± SD, n=3).

**
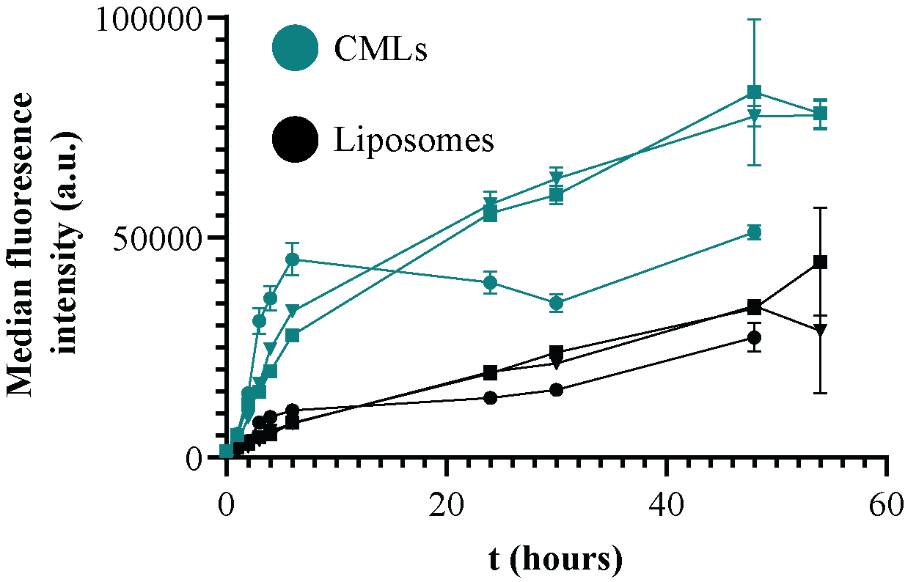
**

**Figure S14.** Median fluorescence intensity of liposomes and CMLs (10 µg mL^-1^) in 10% FBS supplemented medium up to 54 hours measured in MS5 cells using flow cytometry. The results are the average and standard deviation over 3 replicate samples of the median cell fluorescence intensity measured by flow cytometry (mean ± SD, n=3, for 3 replicate experiments). These results are shown again in Figure 8, where all experiment replicates with the optimized liposomes and CMLs are collected in order to show their high reproducibility for multiple membrane extracts and CML batches and in independent experiments (performed with cells at different passage number).


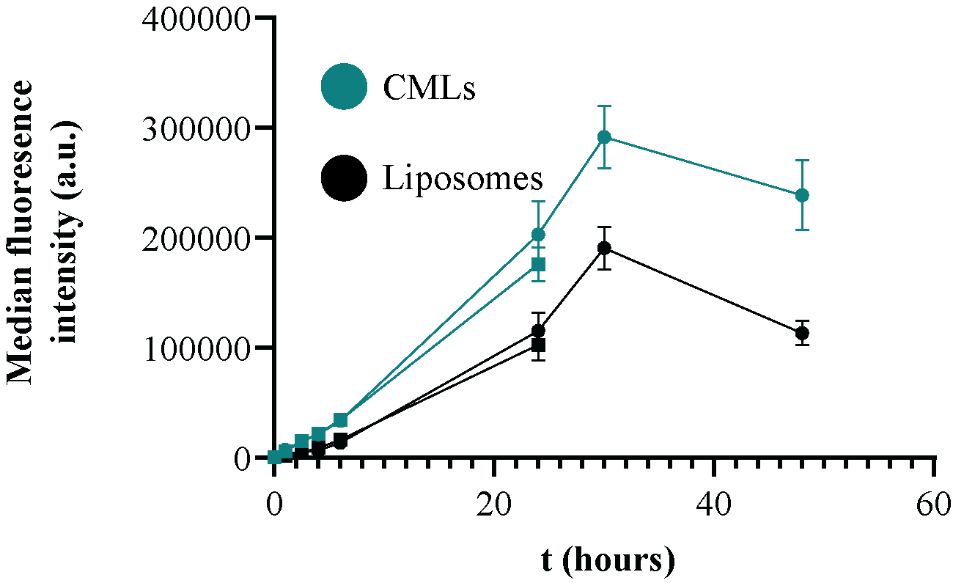


**Figure S15.** Kinetic of uptake of liposomes and CMLs (10 µg mL^-1^) in medium supplemented with 10% FBS in K562 cells up to 48 hours. The results are the average and standard deviation over 3 replicate samples of the median cell fluorescence intensity measured by flow cytometry (mean ± SD, n=3, for 2 replicate experiments). These results are shown again in Figure 8, where all experiment replicates with the optimized liposomes and CMLs are collected in order to show their high reproducibility for multiple membrane extracts and CML batches and in independent experiments (performed with cells at different passage number).
